# Robust impact of tropical Pacific SST trends on global and regional circulation in boreal winter

**DOI:** 10.1038/s41612-025-01192-9

**Published:** 2025-08-27

**Authors:** Joonsuk M. Kang, Rhidian Thomas, Nick Dunstone, Tiffany A. Shaw, Tim Woollings

**Affiliations:** 1 Department of the Geophysical Sciences, The University of Chicago, Chicago, USA; 2 Atmospheric, Oceanic and Planetary Physics, University of Oxford, Oxford, UK; 3 National Centre for Atmospheric Science, University of Reading, Reading, UK; 4 Met Office Hadley Centre, Exeter, UK

**Keywords:** Atmospheric dynamics, Climate-change impacts, Projection and prediction

## Abstract

Evidence has emerged of a discrepancy in tropical Pacific sea surface temperature (SST) trends over the satellite era, where most coupled climate models struggle to simulate the observed La Niña-like SST trends. Here we highlight wider implications of the tropical Pacific SST trend discrepancy for global circulation trends during boreal winter, using two complementary methods to constrain coupled model SST trends: conditioning near-term climate prediction (hindcast) simulations, and pacemaking coupled climate simulations. The robust circulation trend response to constraining the tropical Pacific SST trend resembles the interannual La Niña response. Constraining tropical Pacific SST robustly reduces tropical tropospheric warming, improving agreement with reanalyses, and moderately shifts the zonal-mean jets poleward. It also improves surface air temperature and precipitation trends in ENSO-sensitive regions, such as the Americas, South Asia, and southern Africa. Our results underline the importance of tropical Pacific SST for achieving confidence in multidecadal model projections.

## Introduction

Sea surface temperatures (SST) across the tropical Pacific affect global and regional climate on timescales from seasonal to decadal. Longer-term changes in tropical Pacific SSTs are expected to have substantial implications for the global climate. Over the satellite era, SST trends in this region are characterized by warming in the western Pacific and cooling in the eastern Pacific^1–4^, sometimes described as a “La Niña-like" trend pattern^5,6^. Many different mechanisms have been proposed to explain the observed La Niña-like SST trend, and the physical mechanisms underlying it are debated^4–7^.

The observed La Niña-like SST trend is not captured in most state-of-the-art climate models^3,6,8^. Instead, most of the coupled models participating in the Coupled Model Intercomparison Project Phase 6^9^ simulate an El Niño-like SST trend with greater warming over the eastern Pacific than the western Pacific. After examining hundreds of realizations across CMIP6 models, previous work identified an observation-coupled model discrepancy in tropical Pacific SST trends over the satellite era^3,10^. The SST trend discrepancy is known to impact global mean temperature, radiation, and energy balance^11–14^.

Recent work demonstrated that the observation-coupled model tropical Pacific SST trend discrepancy significantly impacts regional circulations in the Southern Hemisphere during wintertime^15^. In particular, the La Niña-like SST trend involves a Rossby wave teleconnection trend that strengthens the austral winter (June–August, JJA) storm track and weakens the subtropical jet in the South Pacific. In contrast, coupled models capture observed Northern Hemisphere circulation trends in JJA, suggesting a weak impact of the observation-coupled model tropical Pacific SST trend discrepancy^16,17^. The importance of the tropical Pacific SST trend for regional circulation trends during boreal winter (or austral summer, December–February, DJF) has not been closely examined despite emerging circulation trends^18^.

The motivation for studying the impact of the tropical Pacific SST trend during boreal winter is provided by the extensive literature on the impact of interannual El Niño Southern Oscillation (ENSO) variability on regional climate in the season. In particular, the background flow during boreal winter allows ENSO variability to be impactful for both hemispheres^19,20^. In the zonal-mean, the tropical troposphere is cooler, and subtropical jets become weaker in La Niña years^20–22^. This affects Rossby wave momentum fluxes, eddy-driven jet, and storm tracks in the midlatitudes^20,23–25^. Regionally, interannual ENSO variability involves regional circulation changes such as the Pacific-North America pattern^26–28^, and a similar teleconnection impacts the South Pacific and South America^29^. Through teleconnections, the interannual ENSO variability influences remote surface temperature and precipitation during boreal winter over the Americas, South Asia, and southern Africa^29–34^.

The observed, modeled, and theoretical evidence that tropical Pacific SST variability impacts regional climate on interannual time scales motivates an assessment of the impact of tropical Pacific SST trends on regional climate in boreal winter (DJF). However, coupled models cannot easily be used to quantify the impact of tropical Pacific SST trends on regional climate because of the known observation-model SST trend discrepancy^3,6,35^.

Here we use two novel methods that involve constraining coupled model simulations to observations^36^. The first is the hindcast conditioning method^37^, which refers to conditional sampling of interannual hindcast simulations of the DePreSys3 system using the HadGEM3-GC2 model. By randomly sampling one of the 40 ensemble members each year, a large sample of 10,000 long-term trends is generated. The hindcast simulations are sampled at leads of 14–16 months, by which point the spread in tropical Pacific SSTs across the ensemble is sufficient to permit a broad range of multidecadal tropical Pacific SST trends to be generated. The long-term trends are ranked based on their fidelity in simulating observed tropical Pacific SST trends. The top 0.5% with the lowest bias is considered as the Pacific-conditioned (hereafter PacCond) ensemble, and these are compared to the remaining non-conditioned (hereafter NonCond) ensemble. The second method is the pacemaking method^11^, which nudges the tropical east Pacific SST to observations in a fully coupled model. The ensemble of simulations with tropical Pacific pacemaking (hereafter PacPace) is compared with a free-running ensemble that is not nudged (hereafter NonPace), both using the CESM2 model. More details can be found in the Methods section. There are many differences between the methods, including resolution and ensemble size as well as the model and constraining mechanism; despite these differences, several common aspects emerge from the analysis, and we focus on these as robust conclusions. Differences between the two methods are discussed in the final Results section.

Using the hindcast conditioning and pacemaking methods, we quantify the impact of the observation-coupled model tropical SST trend discrepancy on DJF circulation trends over the satellite era. In particular, we address the following questions. What are the robust impacts of improving and capturing the observed tropical Pacific SST trends in the coupled models on the regional circulation and thermodynamic trends? Are the impacts similar to the interannual El Niño Southern Oscillation (ENSO) variability? How do the impacts of capturing tropical SST trends help coupled models capture emerging circulation trends in the observations during DJF? Of particular interest are known trend discrepancies between coupled models and observations, including observed DJF poleward jet trends^18^ that are strong given relatively weak increases in tropical temperatures^38–40^ and upper tropospheric temperature gradients^18^. Lastly, we address how coupled model surface temperature and precipitation trends, in the regions affected by ENSO interannually, improve by capturing tropical Pacific SST trends.

## Results

**Fig. 1:**
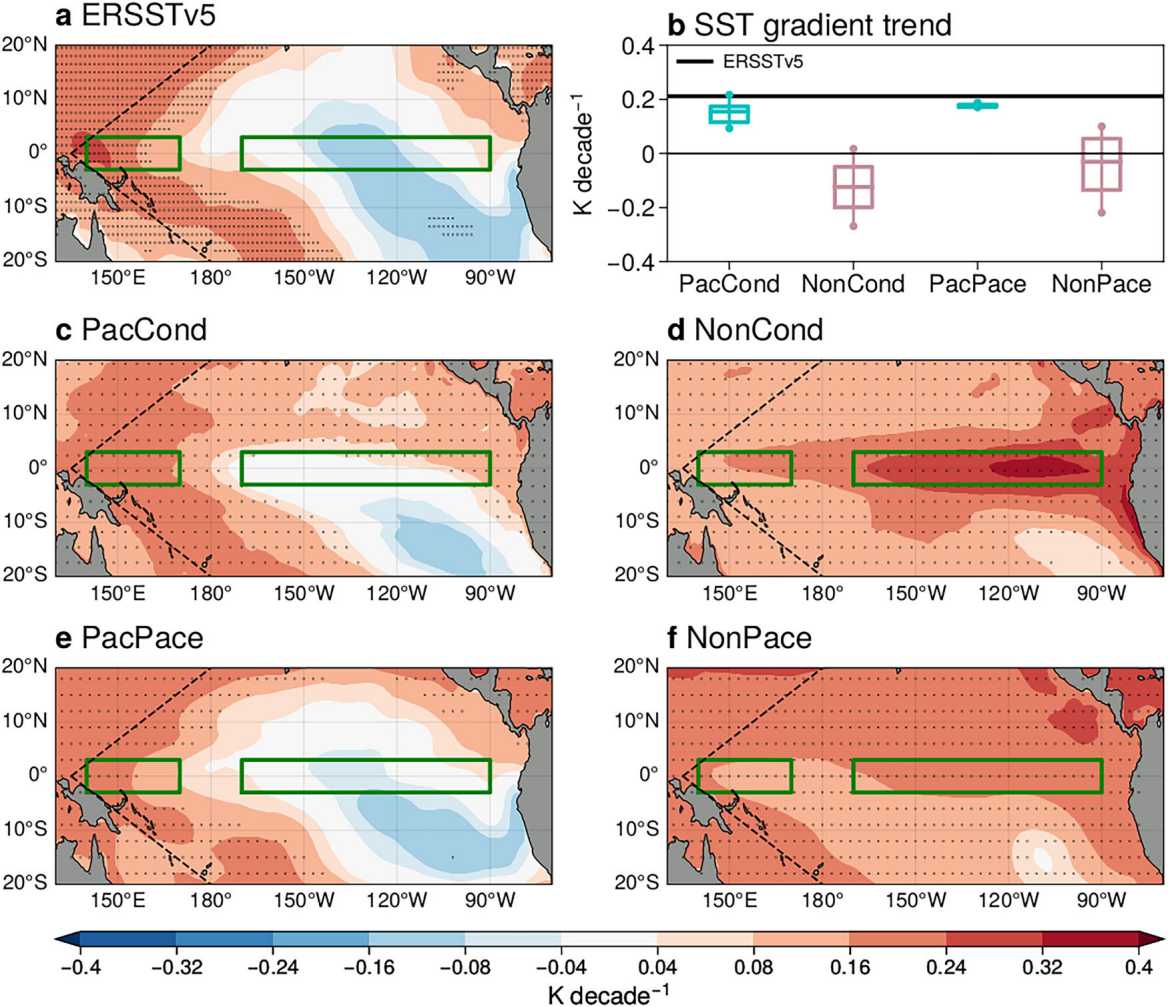
Observed tropical Pacific SST trends are well captured by hindcast conditioning and pacemaking. Tropical Pacific DJF SST trends (1981/82-2018/19) in (**a**) ERSSTv5 and (**c**) PacCond, (**d**) NonCond, (**e**) PacPace, and (**f**) NonPace ensemble-mean. The green boxes indicate where the SST gradient index in (**b**) is calculated following Seager et al.^8^. The black dashed line indicates the pacemaking domain. Stipples indicate statistically significant trends at the 5% significance level. **b** Zonal SST gradient trend in (black) ERSSTv5 and (box and whiskers) the PacCond, NonCond, PacPace, and NonPace ensembles. Boxes represent the interquartile ranges, and whiskers indicate the 10–90% range of the trend distribution. The horizontal line inside each box represents the median trend.

We start by demonstrating the ability of the hindcast conditioning and pacemaking methods in simulating observed tropical Pacific SST trends (Fig. 1). The observed DJF tropical Pacific SST trend from 1981/82 to 2018/19 exhibits warming in the western Pacific and cooling in the eastern Pacific (Fig. 1a), resembling La Niña conditions and strengthening the climatological zonal SST gradient^5,6^ (2.1 K decade^−1^, black line in Fig. 1b). The PacCond ensemble-mean trend also shows warming in the western Pacific and cooling in the eastern (especially southeastern) Pacific (Fig. 1c). Quantitatively, the observed zonal SST gradient trend sits within the 10–90% range of the PacCond ensemble (Fig. 1b). Being nudged to observations, the PacPace ensemble-mean tropical Pacific SST trend also strongly resembles the observed trend (Fig. 1e). All PacPace ensemble members have SST gradient trends very close to the observed trend (1.7–2.1 K decade^−1^). In addition, all members in the Pacific ensembles (PacCond and PacPace) exhibit a strengthening SST gradient (i.e., the cyan whiskers in Fig. 1b are above zero), consistent with the observed trend.

This is in clear contrast with trends in the non-Pacific ensembles (NonCond and NonPace). Most of the members in the NonCond ensemble simulate a weakening zonal gradient (i.e., the pink interquartile box is below zero in Fig. 1b). Similarly, more than half of the members in NonPace ensemble show zonal SST gradient weakening (i.e., the pink median line is below zero in Fig. 1b). Thus, both PacCond and PacPace ensembles show significantly improved SST trends in the tropical Pacific and successfully simulate La Niña-like SST trends, effectively addressing the SST trend discrepancy seen in their unconstrained counterparts.

### Robust impacts of constraining tropical Pacific SST trends

**Fig. 2:**
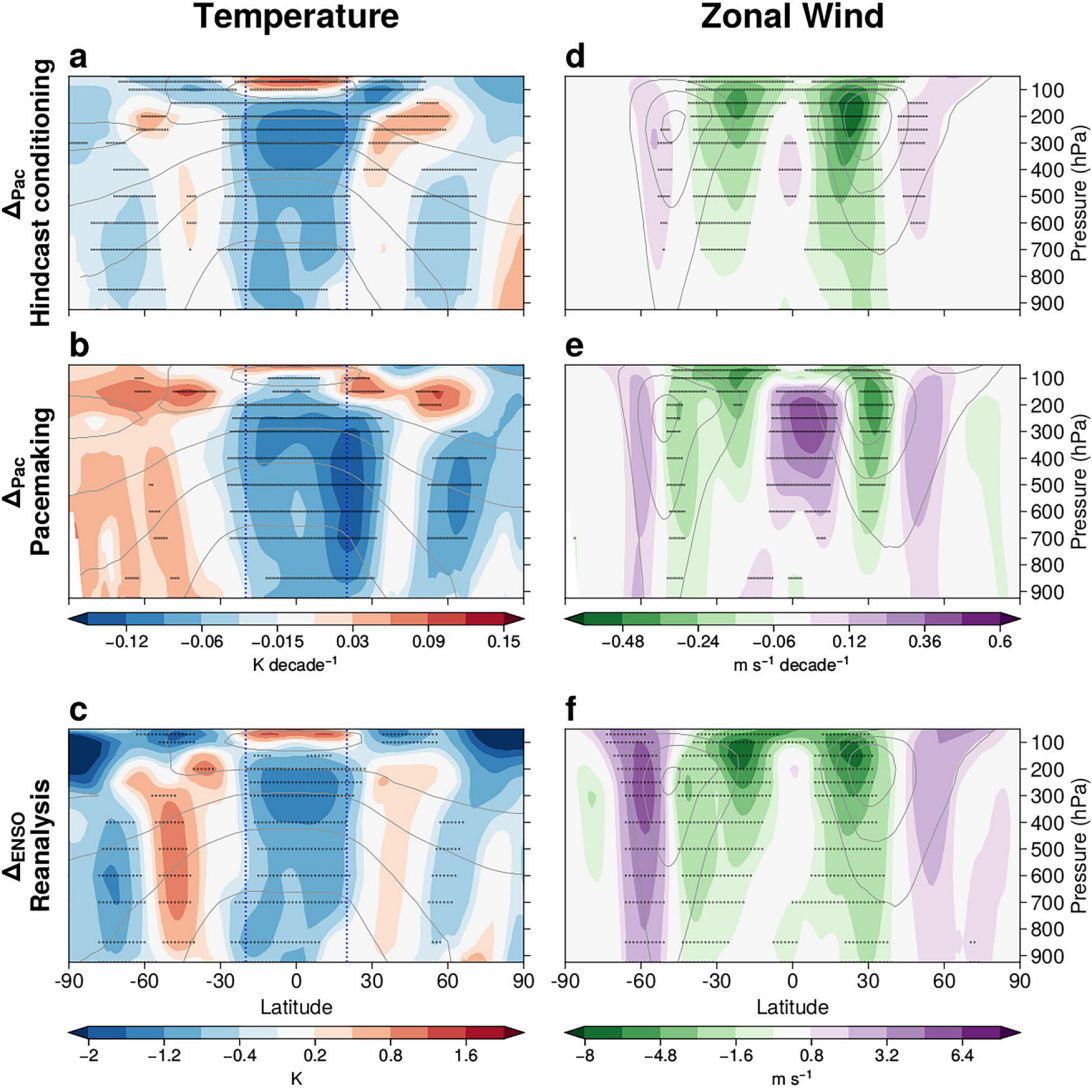
Robust zonal-mean impacts of constraining Pacific SST trends through hindcast conditioning and pacemaking resemble ENSO variability. Impact of constraining tropical Pacific SST trends (Δ_*Pac*_) on the zonal-mean DJF temperature trend (1981/82-2018/19) from (**a**) hindcast conditioning and (**b**) pacemaking. **d**, **e** Similar results to (**a**, **b**), but on the zonal wind trend. **c**, **f** Similar to (**a**, **b**) and (**d**, **e**) but for ENSO response (Δ_*ENSO*_) in reanalysis (ERA5). Stipples indicate statistically significant impacts at the 5% significance level. The blue dashed vertical lines in (**a**) and (**b**) show where the tropical temperature is averaged in Fig. 5.

By constraining the tropical Pacific SST trends, the Pacific ensembles (PacCond and PacPace) simulate trends that are significantly different from their unconstrained counterparts (NonCond and NonPace). The difference in ensemble-mean trends between the Pacific and corresponding non-Pacific ensembles is referred to as the constrained response (labeled Δ_*Pac*_ following Kang et al.^15^). The constrained response quantifies the impact of capturing the observed tropical Pacific SST trend. Focusing on constrained responses that commonly emerge in both methods, the response in zonal-mean temperature is a significant cooling of the tropical troposphere (Fig. 2a, b). The cooling impact is also significant and robust in the Northern Hemisphere (NH) midlatitudes (around 45–75^o^N, Fig. 2a, b).

Following the constrained response in temperature, the constrained response in zonal-mean zonal wind involves a weakening of the subtropical jets in each hemisphere, consistent with the thermal wind response to the cooler tropics (Fig. 2d, e). The NH midlatitude jet robustly strengthens around 45–60^o^N and weakens around 25–45^o^N in the mid-to-lower troposphere (Fig. 2d, e). The SH midlatitude jet also intensifies around 50–65^o^N and weakens around 30–40^o^N. Thus, capturing tropical Pacific SST trends is associated with a dipole trend of the zonal-mean zonal wind in both hemispheres. This robust response is consistent with the connection between tropical decadal variability and SH jet during DJF^41^.

We also compare the constrained response to the interannual ENSO variability in reanalysis, since tropical Pacific SST trends in the Pacific ensembles (Fig. 1c and e) and non-Pacific ensembles (Fig. 1d, f) resemble La Niña and El Niño, respectively. The reanalysis ENSO response, Δ_*ENSO*_, is calculated as the composite difference between 5 La Niña and 6 El Niño years (see Methods). Although with different units, the zonal-mean temperature and zonal wind ENSO responses in the reanalysis exhibit similar patterns to the constrained response for each method (Fig. 2c, f, see also Fig. S1 for ENSO in PacCond and PacPace). This suggests that the mechanisms underlying the zonal-mean constrained response (Δ_*Pac*_) can be understood in line with the interannual ENSO response (Δ_*ENSO*_) studied in previous work^20,22^.

**Fig. 3:**
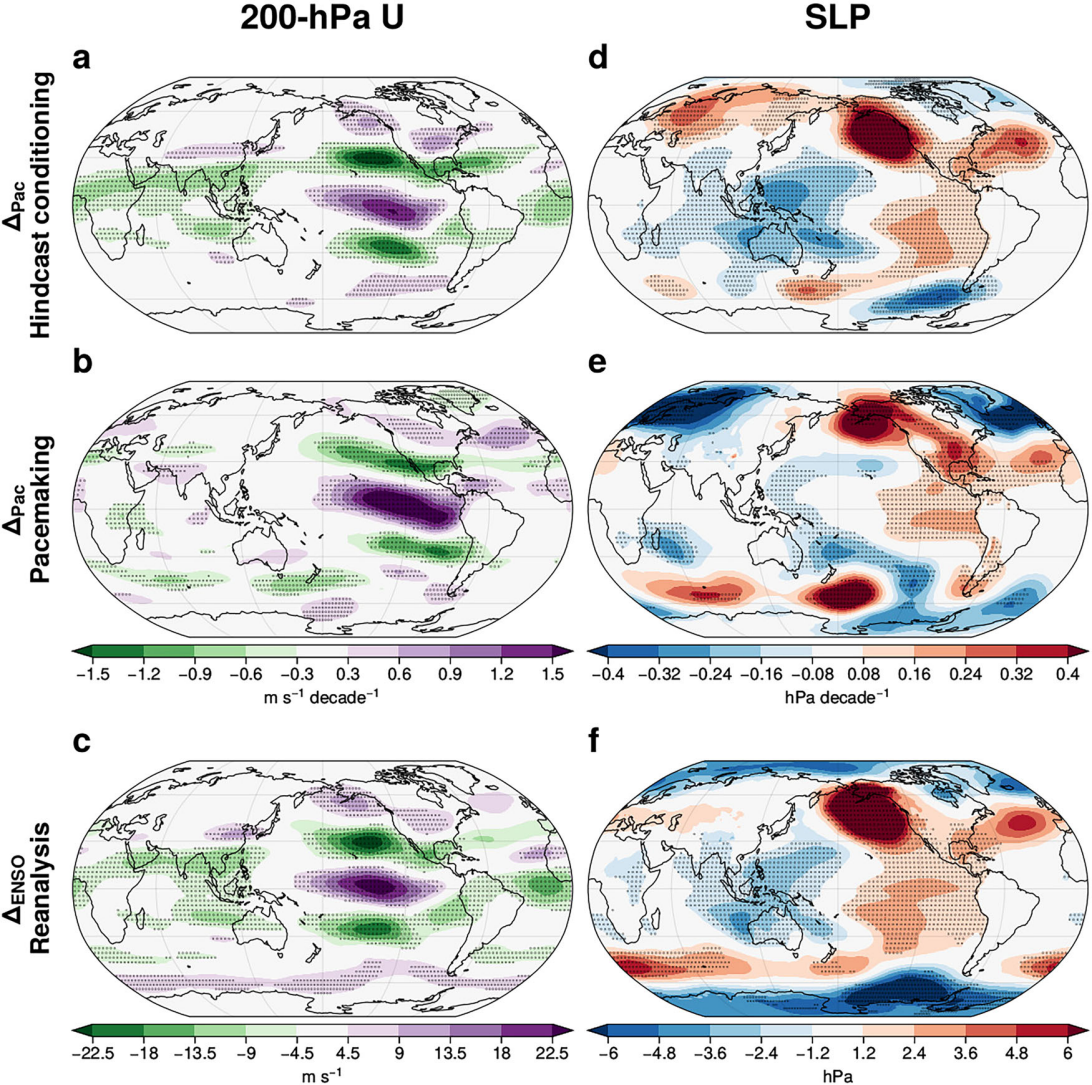
Robust regional circulation impacts of constraining Pacific SST trends through hindcast conditioning and pacemaking resemble ENSO variability. Impact of constraining tropical Pacific SST trends (Δ_*Pac*_) on the DJF 200-hPa zonal wind trend (1981/82-2018/19) from (**a**) hindcast conditioning and (**b**) pacemaking. **d**, **e** Similar results to (**a**, **b**), but on the SLP trend. **c**, **f** Similar to (**a**, **b**) and (**d**, **e**) but for ENSO response (Δ_*ENSO*_) in reanalysis (ERA5). Stipples indicate statistically significant impacts at the 5% significance level.

Regionally, the constrained response in zonal wind is most pronounced over the eastern Pacific and the Americas for both methods (Fig. 3a, b). The constrained response in upper tropospheric (200-hPa) wind in the tropical eastern Pacific (Fig. 3a, b) is a westerly trend, consistent with the strengthening of the Walker circulation and zonal SST gradient^3^ (Fig. 1e, Fig. 3d, e). The subtropical jet weakens in both hemispheres in the eastern Pacific with strengthening westerlies at higher latitudes (Fig. 3a and b), indicating Rossby wave teleconnection trends^15^. The constrained response over the Pacific suggests an enhanced poleward shift, consistent with recent work which showed a modest contribution of Pacific SSTs to the northward shift of the North Pacific jet^42^. Similar patterns of responses are identifiable in the lower troposphere (850 hPa, Fig. S2), consistent with the barotropic structure in Fig. 2d, e.

Across the tropical Pacific, the positive constrained response in SLP in the eastern Pacific shows a strengthening of the Walker circulation (Fig. 3d, e). Consistent with the Rossby wave teleconnection trends, SLP strengthens to the northwest and southeast of North America in both methods (Fig. 3d, e), resembling the Pacific-North American pattern^26^. Over the Atlantic, the constrained responses in SLP exhibit a positive North Atlantic Oscillation-like pattern, consistent with the late-winter response to La Niña^43,44^, while the response is stronger in the PacPace ensemble. Regional weakening of SLP over the Indian Ocean east of Africa is also found (Fig. 3d, e). In the SH mid-latitudes, the constrained response in SLP exhibits a wave pattern, which is likely related to tropical diabatic heating^45^. As before, the constrained responses (Δ_*Pac*_) in zonal winds and SLP resemble the ENSO response (Δ_*ENSO*_, Figs. 3c, f and S3).

**Fig. 4:**
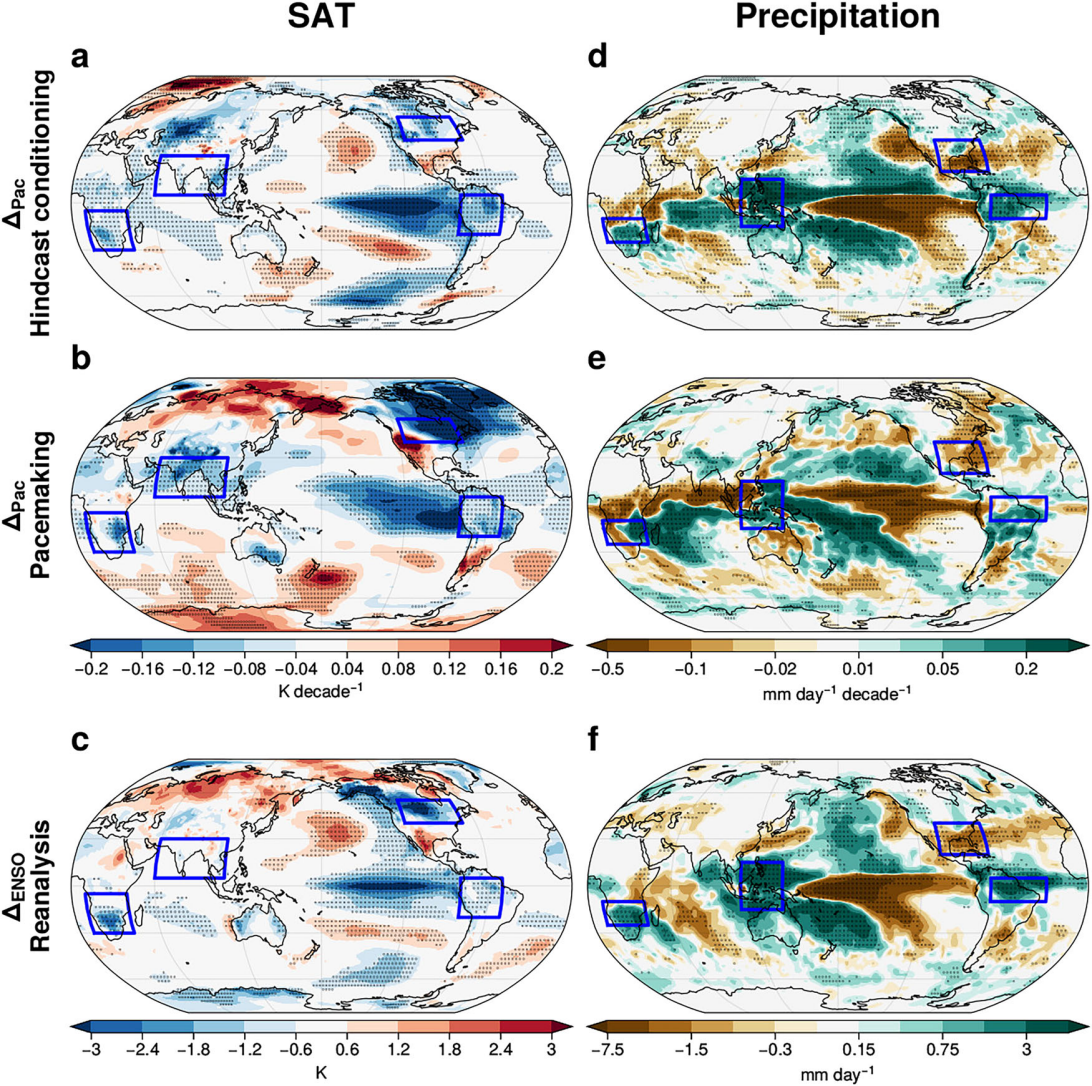
Robust surface impacts of constraining Pacific SST trends through hindcast conditioning and pacemaking resemble ENSO variability. Impact of constraining tropical Pacific SST trends (Δ_*Pac*_) on the DJF SAT trend (1981/82-2018/19) from (**a**) hindcast conditioning and (**b**) pacemaking. **d**, **e** Similar results to (**a**, **b**), but on the precipitation trend. **c**, **f** Similar to (**a**, **b**) and (**d**, **e**) but for ENSO response (Δ_*ENSO*_) in reanalysis (ERA5 for SAT and GPCP for precipitation). Stipples indicate statistically significant impacts at the 5% significance level. The blue boxes indicate regions significantly affected by Δ_*Pac*_ and Δ_*ENSO*_ discussed in the text.

The constrained responses in regional circulation are connected with trends in surface impacts. Constraining tropical Pacific SST trends leads to cooling impacts over the eastern tropical Pacific and low-latitude South America, consistent with cooler eastern Pacific SST (Fig. 4a, b). Cooling impacts are also found over midlatitude North America for both methods (Fig. 4a, b) and are likely related to the northerly trend by the strengthened SLP trend to the west (Figs. 3d, e and S2c, d). The constrained responses in SAT also involve cooling over South Asia and southern Africa (Fig. 4a, b). The cooling impacts in southern Africa are associated with southerly trends (Fig. S2c, d). Overall, these constrained responses (Δ_*Pac*_) in SAT can be anticipated from the interannual ENSO responses (Δ_*ENSO*_) in reanalysis (Fig. 4c). It is also worth noting that SAT responses over the extratropical ocean are also consistent with responses in SST (Fig. S4). This suggests that the extratropical SST trend responses are also driven by atmospheric circulation, consistent with interannual ENSO responses^46^.

Lastly, precipitation trends are also impacted by constraining the tropical Pacific SST trends. Across the tropical east Pacific, drying (negative precipitation) trends are found broadly, whereas wetting (positive precipitation) trends are found in the Maritime Continent (Fig. 4d, e). Over the Americas, drying impacts are found over the southeast of North America, whereas wetting impacts dominate the low-latitude South America (Fig. 4d, e). These constrained responses over the Americas are consistent with recent work using statistical adjustment^47^. In the southeast of South America, there are robust drying trends (Fig. 4d, e). Over Africa, drying trends are found near the equator and wetting trends to its south (Fig. 4d and e). Similar to most of the impacts above, the Δ_*Pac*_ in precipitation is consistent with Δ_*ENSO*_ in observations (Fig. 4f). Moreover, the concomitant responses in SAT and precipitation suggest that land moisture and cloud interactions^31^ during ENSO are also operating on multidecadal time scales.

In short, the responses to constraining tropical Pacific SST trends (Δ_*Pac*_) are robust across both methods. The pattern of the multidecadal trend response resembles the interannual La Niña minus El Niño response (i.e., Δ_*Pac*_ resembles Δ_*ENSO*_), establishing that interannual teleconnection theory is a useful guide for impacts on longer timescales.

### Improvements in large-scale trends

**Fig. 5:**
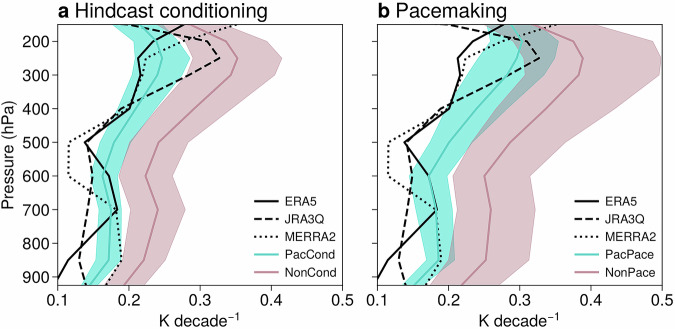
Constraining Pacific SST trends improves coupled model tropical temperature trends. **a** Vertical profile of zonal-mean DJF tropical temperature trends (1981/82–2018/19) in reanalyses (black lines), and the PacCond (cyan) and NonCond (pink) ensemble-means. The shadings represent 10–90% of the ensemble trend distributions. **b** Similar results for the PacPace (cyan) and NonPace (pink) ensembles. Tropical means are calculated over 20^o^S–20^o^N (see the blue vertical lines in Fig. 2a, b).

In this section, we compare the constrained Pacific ensembles to reanalyses, highlighting improvements in large-scale atmospheric trends that result from constraining the tropical Pacific SST trend. A notable example is the temperature trends in the tropical free troposphere, shown in Fig. 5. The tropical free troposphere exhibits slower warming than the subtropics, particularly over the eastern Pacific and Atlantic (Fig. S6a). Coupled models typically overestimate the rate of satellite-era warming in the tropics compared to trends inferred from observations and reanalyses during DJF as well as other seasons^38–40,48,49^. This is also found in the non-Pacific ensembles (NonCond and NonPace, Fig. 5). While considerable spread is seen in the reanalysis trends, all three products lie almost entirely outside the 10–90% range of trends in the non-Pacific ensembles throughout the troposphere (i.e., the black lines lie outside the pink shading).

As shown in the previous section, constraining the tropical Pacific SST trend leads to smaller temperature trends in the zonal-mean tropical troposphere (Fig. 2a, b). This brings the models into better agreement with the reanalysis trends (cyan shading in Fig. 5). Quantitatively, at least one reanalysis product is within the 10–90% range of Pacific model trends at all levels besides 500hPa. By contrast, below 250hPa, none of the reanalysis trends are within the 10–90% range of trends in the non-Pacific ensembles. Regionally, the midtropospheric cooling response is strongest over the Pacific sector in both ensembles (Fig. S5), corresponding to the region of slowest warming in the observed trend (Fig. S6a). Previous work has shown that, in coupled models, weak mid-tropospheric temperature trends are associated with patterns of SST variability resembling the negative phase of the Pacific Decadal Oscillation^40,50^. Figure 5 quantitatively shows that the tropospheric trend discrepancy between models and reanalyses is substantially reduced in models that capture observed SST trends in the tropical Pacific.

**Fig. 6:**
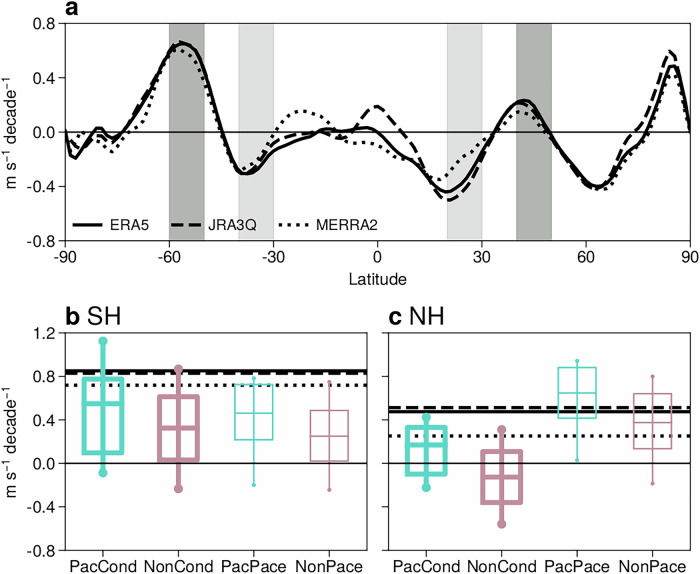
Constraining Pacific SST trends improves poleward jet shift trends in coupled models. **a** DJF 500h-Pa zonal-mean zonal wind trends (1981/82-2018/19) in reanalyses. **b**, **c** Distribution of DJF jet shift trends (1981/82-2018/19) in the (**b**) SH and (**c**) NH, respectively, using the zonal indices of Woollings et al.^18^. Positive trends correspond to poleward jet shifts. Boxes indicate the interquartile range and median trend, while whiskers represent 10–90% of the trend distribution. Thicker lines indicate where the ensemble-mean trends in the Pacific and non-Pacific ensembles are significantly different at the 5% significance level, using a two-tailed Student’s t-test. Reanalysis trends are shown by the black horizontal lines. Light and dark gray shading in (**a**) indicates the equatorward and poleward latitude bands used to calculate the zonal wind indices.

Due to cooler tropical temperature trends, meridional temperature gradient trends weaken, and the weakening subtropical jet trends are better simulated in the Pacific than the non-Pacific ensembles (Fig. S7). In the midlatitudes, poleward shifts of the DJF zonal-mean jets are seen in reanalyses over the satellite era (Fig. 6a). In the NH, the trends over the North Pacific contribute most to the zonal mean trend (Fig. S6b). In the SH, the poleward shift is found across most longitudes (Fig. S6b). While the poleward shift of the zonal-mean jet is consistent with coupled model trends, previous work noted coupled models tend to simulate poleward shifts in conjunction with larger strengthening of upper-level meridional temperature gradients than reanalysis, especially in DJF^18^.

We revisit the poleward zonal-mean jet shifts in reanalysis and the Pacific and non-Pacific ensembles using the zonal wind indices of Woollings et al.^18^. The light and dark gray shading in Fig. 6a shows the latitude bands used to calculate the zonal indices, situated on the equatorward and poleward flanks of the climatological wind maximum in each hemisphere. The index measures meridional zonal-mean jet shifts as the trend difference between the poleward and equatorward latitude bands, with positive values corresponding to poleward shifts. In the SH, jet shifts in the non-Pacific ensembles are similar to each other, with most of the ensemble members showing a weak poleward shift (i.e., the pink boxes are above zero, Fig. 6b). The non-Pacific ensembles differ more in the NH, with the NonPace ensemble tending to favour more poleward shifts than the NonCond ensemble and no overlap between the interquartile ranges of trends (pink boxes, Fig. 6c).

In both hemispheres, constraining the tropical Pacific trend leads to stronger poleward jet shifts (about 0.18 m s^−1^ decade^−1^ in zonal index), consistent with the ensemble-mean trend response (Fig. 2d, e). In the SH, the ensemble-mean zonal index trends increase by 50–64% by constraining the tropical Pacific SST trend (Fig. 6b). In the NH, the ensemble-mean zonal index trend is about 55% greater in PacPace than NonPace ensemble, and it changes sign between PacCond and NonCond (Fig. 6c). However, the effect size (i.e., the difference in median trends) is modest compared to the spread between ensemble members, and the constrained response is not statistically significant in either hemisphere in the PacPace ensemble. In the SH, the 10−90% range of trends in the Pacific ensembles spans zero for both models, while the poleward shifts in reanalyses remain towards the upper edge of the model distributions (particularly for PacPace).

In addition to the spread of trends within each model, differences between the models also have a larger effect than capturing the tropical Pacific trend, particularly in the NH. The NH zonal-mean jet shifts in the NonPace ensemble are generally greater than those in the NonCond ensemble, and even those in the PacCond ensemble (Fig. 6c), despite the generally poor tropical Pacific SST trends in NonPace (Fig. 1b, f). In summary, while constraining the tropical Pacific SST trend does improve zonal-mean jet trends on average, trends in any pair of model realisations will in general differ more due to factors external to the tropical Pacific, including intrinsic atmospheric variability and structural differences between models. While the focus above is on the zonal-mean, Patterson et al.^42^ similarly concluded that tropical Pacific SST trends are unable to fully explain the much larger northward shift of the winter North Pacific jet in observations than in coupled models.

### Improvements in surface impact trends

**Fig. 7:**
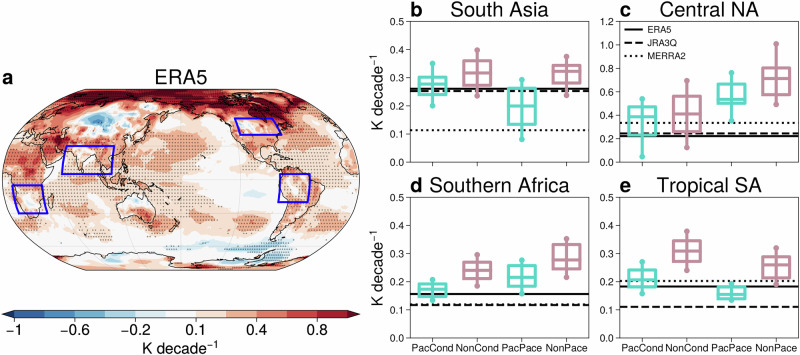
Constraining Pacific SST trends improves coupled model surface air temperature trends over land. **a** DJF SAT trends (1981/82-2018/19) in ERA5. Statistically significant trends at the 5% significance level are stippled. The blue boxes represent regions impacted by Pacific SST improvement. Distribution of land SAT trends in the PacCond, NonCond, PacPace, and NonPace ensembles in (**b**) South Asia, (**c**) central North America, (**d**) southern Africa, and (**e**) tropical South America. Boxes indicate the interquartile range and median trend, while whiskers represent 10–90% of the trend distribution. Note that only trends over land grid points are quantified in panels (**b**–**e**). Thicker lines indicate where the ensemble-mean trends in the Pacific and non-Pacific ensembles are significantly different at the 5% significance level, using a two-tailed Student’s t-test. Reanalysis trends are shown by the black horizontal lines.

We now investigate improvements in regional surface impact trends that result from constraining the tropical Pacific SST trend. Figure 7b–e shows land SAT trends in four regions chosen following the ENSO response (blue boxes in Fig. 4a–c and Fig. 7a). SAT trends are consistently reduced in the Pacific ensembles (cyan, Fig. 7b–e), bringing the coupled model trends closer to the reanalyses. As a consequence, two or more reanalysis trends are within the Pacific ensemble spread in South Asia (Fig. 7b). In central North America, the improvement is modest for hindcast conditioning (i.e., the difference between PacCond and NonCond is small), but pacemaking brings coupled model trends significantly closer to reanalysis trends (Fig. 7c). In southern Africa, all reanalysis trends are outside the 10–90% range of the non-Pacific ensembles, whereas the ERA5 trend is within the range of both Pacific ensembles (Fig. 7d). In tropical South America, which is adjacent to the targeted tropical Pacific region, the spread of trends in the Pacific ensembles is narrow, with the constrained models simulating land SAT trends close to the reanalyses (Fig. 7e). Thus, capturing the Pacific SST trend helps the coupled models capture SAT trends over the land regions impacted by La Niña and El Niño.

**Fig. 8:**
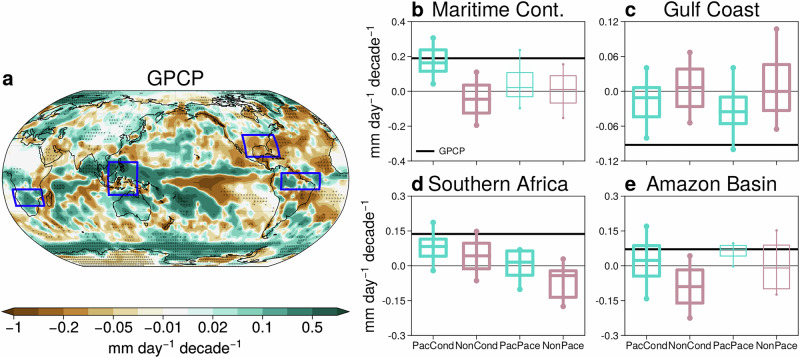
Constraining Pacific SST trends improves coupled model precipitation trends over land. **a** DJF precipitation trends (1981/82-2018/19) in GPCP. Statistically significant trends at the 5% significance level are stippled. The blue boxes represent regions impacted by Pacific SST improvement. Distribution of land precipitation trends in the PacCond, NonCond, PacPace, and NonPace ensembles in (**b**) Maritime Continent, (**c**) Gulf Coast, (**d**) Southern Africa, and (**e**) Amazon Basin. Boxes indicate the interquartile range and median trend, while whiskers represent 10–90% of the trend distribution. Note that only trends over land grid points are quantified in (**c**–**e**), while (**b**) Maritime Continent includes trends over ocean grid points. Thicker lines indicate where the ensemble-mean trends in the Pacific and non-Pacific ensembles are significantly different at the 5% significance level, using a two-tailed Student’s t-test. GPCP trends are shown by the black horizontal lines.

Constraining SST trends in the tropical Pacific has a leading-order effect on regional precipitation trends over land. Figure 8b–e shows precipitation trends in four regions that are impacted by the tropical Pacific on interannual ENSO responses and which show a robust constrained response to the tropical Pacific SST trend (the blue boxed regions in Fig. 4d–f). Over the Maritime Continent, the observed positive precipitation trend is not captured by the non-Pacific ensembles (pink, Fig. 8b). The sign of the trends in the non-Pacific ensembles is also not robust (i.e., the pink boxes cross zero, Fig. 8b). Significantly more positive trends are seen in PacCond than in the NonCond ensemble, which captures the observed trend well. While trends in PacPace and NonPace ensembles are not significantly different in a statistical sense, the observed trend is captured by the PacPace but not by the NonPace ensemble.

Similarly, in Southern Africa, significantly more positive precipitation trends are seen in the Pacific ensembles than their non-Pacific counterparts (Fig. 8d). This moves the models closer to the observed positive trend. Most NonPace ensemble members show a drying trend over Southern Africa, whereas the median trend in PacPace ensemble is weakly positive. While the NonCond ensemble performs better than the NonPace ensemble, agreement with the observed trend is again improved by capturing the Pacific SST trend in the PacCond ensemble.

Precipitation trends in the non-Pacific ensembles are weak and sometimes positive over land in the Gulf Coast region, with the median trends being nearly zero (i.e., the pink median lines are near zero, Fig. 8c). When the tropical Pacific SST trend is captured, the regional trends are significantly more negative in both models (cyan, Fig. 8c), and the observed trend is captured by the PacPace ensemble.

Finally, over the Amazon Basin, the observed positive precipitation trend over land is underestimated in the NonCond ensemble (Fig. 8e). Constraining the Pacific SST trend is necessary in this model in order to capture observed precipitation trends (PacCond, Fig. 8e). While the observed trend is already captured by NonPace, the model supports a wide range of trends of both signs. In contrast, trends in PacPace ensemble are tightly bunched around the observed trend (Fig. 8e), indicating the strong control exerted by the Pacific SST trend on the Amazonian precipitation trend.

### Comparison of SST constraining methods

We have thus far focused on the robust impacts of constraining the tropical Pacific SST trends in boreal winter. However, there are some differences between the impacts quantified using the two methods. For example, the zonal-mean cooling response over the NH subtropics and the zonal-mean upper-level westerly response at the equator are found in CESM2 pacemaking but not in DePreSys3 hindcast conditioning (compare Fig. 2a,d and b,e). Regionally, these differences are related to differing responses outside the Pacific sector (Figs. 3 and S5). These differences could arise due to differences in the SST trend-constraining methods (hindcast conditioning and pacemaking) and/or the differences in models.

The hindcast trends in PacCond are formed by concatenating discrete ENSO events, and hence slow feedbacks that couple different years, such as changes in ocean circulation or the accumulation of stratospheric water vapour^51^, are neglected. Also neglected are lagged teleconnections to the tropical North Atlantic and Indian oceans, which persist through the boreal spring and boreal summer following ENSO events, respectively^52,53^. On the other hand, the PacPace simulations are continuous integrations that evolve freely outside the tropical Pacific region, and can hence probe the influence of slow feedbacks from the tropical Pacific SST trend. Lagged teleconnections could therefore explain the stronger tropical North Atlantic cooling response in PacPace than PacCond (c.f. Fig. 4a, b) and the correspondingly weaker Amazon basin precipitation response (Fig. 4d–e). An additional difference is that only the tropical east Pacific is nudged in PacPace, whereas the tropics evolve freely in the ENSO events that form the PacCond sequence.

One way to quantitatively understand the differences is to repeat the hindcast conditioning using CESM2. Thus, we performed hindcast conditioning using the simulations from the Seasonal-to-Multiyear Large Ensemble prediction system (SMYLE^54^), which is a hindcast ensemble using CESM2 (Fig. S8). In SMYLE, the impact of constraining tropical Pacific SST trends on the zonal mean temperature and zonal wind trends is more similar to Δ_*Pac*_ from hindcast conditioning than from pacemaking (cf. Figs. 2 and S9). Thus, it is likely that the zonal mean response differences between DePreSys3 hindcast conditioning and CESM2 pacemaking are due to the difference in SST trend-constraining methods (that only the tropical east Pacific is nudged in the PacPace ensemble) rather than the difference in models. Nevertheless, it is worth noting that we find similar robust responses to those identified in DePreSys3 hindcast and CESM2 pacemaking in SMYLE hindcast (Figs. S8 and S9), further building confidence in our conclusions.

## Discussion

It is well established that the current generation of coupled models struggles to simulate observed SST trends in the tropical Pacific^3^. Less attention has focused on the consequences of the tropical Pacific SST trend discrepancy for regional circulation and thermodynamic trends, because of the difficulty in using coupled models to investigate them. We used two novel and complementary methods to constrain tropical Pacific SST trends in coupled models: hindcast conditioning, which samples a very large ensemble of hindcast trends based on their representation of Pacific SST trends, and pacemaker experiments, where model SST anomalies in the tropical Pacific are relaxed to observed values.

The circulation response to constraining the tropical Pacific SST trend in boreal winter closely resembles the interannual response to La Niña in the models and reflects the fast atmospheric response to tropical Pacific SST variability. The tropics cool in the zonal-mean and the subtropical jets weaken, and a dipole trend is found across midlatitudes. Regionally, the Pacific Walker circulation strengthens, the Aleutian Low weakens, and the jet shifts poleward over North America, consistent with the Pacific-North American teleconnection^26,55^. The North Atlantic response projects onto positive NAO, though the low pressure response is stronger and extends further into Eurasia in the PacPace ensemble.

We also show that constraining the tropical Pacific trend improves the coupled models’ agreement with trends in observations and reanalyses. Reanalysis tropical tropospheric temperature trends mostly fall within the 10−90% range of trends in the Pacific ensembles, whereas the non-Pacific ensembles overestimate tropical warming. This reduces meridional temperature gradient trends and improves coupled model simulation of subtropical jet weakening trends. Constraining the tropical Pacific SST trend also leads to ensemble-mean jet shift trends that are 50–64% more poleward in the SH and 50% in the NH. However, variability outside the tropical Pacific and structural differences between models remain key factors in modeled jet trends over this period. Nevertheless, with cooler tropical upper-level temperature trends, the Pacific ensembles reduce the combined meridional temperature gradient-poleward jet shift discrepancy identified in coupled models by Woollings et al.^18^. We also show that capturing the tropical Pacific SST trend is necessary for reproducing observed regional temperature and precipitation trends over Southern Africa, the Maritime Continent, and parts of the Americas.

The hindcast conditioning and pacemaking methods used to constrain SST trends are complementary. The hindcast conditioning approach leverages a very large ensemble to sample rare coupled model trends: although only a small fraction of the 10,000 members come close to capturing the observed trend, the resulting conditioned subset is still large enough to allow for robust statistics. Achieving a conditioned subset of comparable size by sampling free-running coupled simulations would require considerable computational expense, whereas the hindcasts approach efficiently exploits existing resources to build a very large ensemble of plausible trends. While slow feedbacks are neglected in the PacCond response, energy is conserved and full coupling is retained within each hindcast simulation. An additional benefit is the relatively high horizontal and vertical resolution of DePreSys3, and its skill in representing teleconnections from the tropical Pacific as verified against observations^56^; through its frequent re-initialisation from reanalysis, mean state biases are also minimised. While the PacPace ensemble is smaller, it isolates the causal effect of the tropical Pacific SST trend by directly perturbing the model SST field. It also permits slow feedbacks that couple the responses between successive years, unlike in PacCond.

The methods used here can be used to quantify the impact of tropical Pacific SST trends in other seasons. Investigating the impact of constraining tropical Pacific SST trends during June-July-August (JJA) using the two methods reveals strong and robust circulation responses over the South Pacific sector (Fig. S10). Both methods indicate a pressure dipole response consistent with subtropical jet weakening and midlatitude jet strengthening (see also Fig. 6 in Kang et al.^15^). A drying response is also seen over the coast of Chile (Fig. S10g, h), consistent with work linking South Pacific pressure trends to the recent Chilean multi-year drought^57^. Wetting responses are also robustly found over central Africa, India, and Central America.

In contrast, the NH response is generally weak: hindcast conditioning shows no circulation response anywhere in the NH (Fig. S10a, c), and pacemaking shows a moderate response over the North Pacific but weak signals downstream, consistent with the weaker waveguiding mean state in NH summer (Fig. S10b, d). This aligns with recent findings that coupled models capture the NH summertime circulation trend despite the tropical Pacific SST trend discrepancy^17^. The non-robust NH circulation trend responses from the two methods also suggest that processes other than tropical Pacific SST trends^58^ can be important for the stationary wave trends. The response from hindcast conditioning is weak since shorter lead times are used for JJA (see Methods), and the SST trend difference between PacCond and NonCond is small.

The results in this paper have been interpreted through teleconnections from the tropical Pacific that are well established on seasonal timescales, and which appear to also hold in projections of future climate^59^. However, with only a small pool of La Niña and El Niño events in the observed record, even these relationships could be considered uncertain, particularly outside the Pacific and North American sectors^28^. Unlike the interannual La Niña and El Niño composites, which isolate the influence of the tropical Pacific, the single observed trend also contains contributions from outside the Pacific, as well as the anthropogenically forced trend.

We also expect that constraining tropical Pacific SST trend would imprint on extratropical SSTs^60^—both via the atmospheric circulation (both methods) and potentially through changes to ocean circulation (only in pacemaking). Examining the extratropical SST trends (Fig. S4), the response over the Pacific is largely similar in both models and resembles the SST response to ENSO variability^46^, suggesting the importance of the atmospheric circulation response. Differences are somewhat larger over other extratropical oceans, potentially reflecting the importance of ocean processes and slower feedbacks.

Consistent with recent works^15,61^, this study has quantified the significant impact of recent trends in the tropical Pacific SST, in which the zonal SST gradient has strengthened. This highlights the importance of understanding the physical mechanisms underlying the tropical Pacific SST trends^4,6^. Coupled models project a future reversal of this trend, leading to a weakened east-west gradient. However, confidence in these projections is limited by the failure of models to capture the recent trend. If present trends continue, our findings suggest that models will continue to struggle to capture trends elsewhere in the climate system, from tropical warming to regional temperature and precipitation trends.

## Methods

### Observational data

We use various observational data to quantify the trends from 1981/82 to 2018/19 during DJF. SST data is used from ERSSTv5^62^. Trends in surface temperature and atmospheric circulation are quantified from three reanalysis products: MERRA2^63^, ERA5^64^, and JRA3Q^65^. For precipitation trends, we use the GPCP monthly products version 2.3^66^. The later GPCP version 3.2 dataset^67^ starts from 1983 and therefore is not considered here. Nevertheless, previous work showed that the trends in the two versions are largely similar^67^. Trends are quantified using least-squares linear regression.

### Pacemaking: CESM2 Pacific Pacemaker simulations

We use pacemaking to constrain the tropical Pacific SST trends in coupled simulations. Specifically, we use the CESM2 tropical Pacific pacemaker ensemble (see https://www.cesm.ucar.edu/working-groups/climate/simulations/cesm2-pacific-pacemakerfor details, also Kang et al.^15^). The Pacific pacemaker ensemble (PacPace) is a 10-member initial condition ensemble using the CESM2 model^68^. The simulations have a nominal 1^o^ horizontal resolution both in the atmosphere and ocean. They are run from 1880 to 2019 and are forced with the CMIP6 historical (until 2014) and SSP3-7.0 (after 2015) radiative forcings. The simulations are fully coupled except in the regions where SST anomalies (relative to 1880–2019 climatology) are nudged to observed anomalies from ERSSTv5^62^ (black dashed line in Fig. 1). Thus, by construction, it captures the observed tropical Pacific SST trends.

The impact of constraining tropical Pacific SST trend is quantified by comparing the PacPace ensemble to the CESM2 large ensemble simulations^69^. The CESM2 large ensemble is a 50-member initial condition ensemble that has the same horizontal resolution and is forced with the same radiative forcing as the PacPace ensemble. The key difference compared to the PacPace ensemble is that pacemaking is not applied to the tropical Pacific with SST freely evolving in the CESM2 large ensemble. The CESM2 large ensemble is thus termed the NonPace ensemble. The NonPace ensemble does not capture the observed tropical Pacific SST trend (Fig. 1b, f). Differences in the mean of the trend distributions between the PacPace and NonPace ensembles are tested using a Student’s t-test at the 5% significance level.

### Hindcast conditioning: DePreSys3 hindcast simulations

We also use hindcast conditioning to constrain the tropical Pacific SST trends. We utilize interannual hindcast simulations from the third iteration of the Met Office decadal prediction system^56,37^ (DePreSys3), which uses the HadGEM3-GC2 coupled model^70,71^. The HadGEM3-GC2 model has a horizontal resolution of approximately 0.83^o^ × 0.56^o^ in the atmosphere and 0.25^o^ in the ocean. The initial conditions of the hindcast simulations are derived from ERA-Interim^72^ reanalysis for atmospheric winds and temperature, MOSORA^73^ for ocean temperature and salinity, and HadISST^74^ for sea ice. The simulations are forced with CMIP5 historical (until 2005) and RCP4.5 (after 2006) radiative forcings.

A 40-member hindcast simulation ensemble is initialised on the 1st of November in each year from 1980 to 2022, and is run for 17 months. We use data from months 14 to 16 of each simulation (the second DJF) to ensure sufficient spread in simulated tropical Pacific SST. This results in 40 data points each year. We focus on DJF from 1981/92 to 2018/2019, the overlapping period with the PacPace ensemble. Then, we create 10,000 bootstrapped timeseries by randomly selecting one of the 40 members each year following Thomas et al.^37^. Each timeseries is referred to as a trend member. Trend members for JJA (Fig. S10) are created using data from months 8 to 10 of each simulation; thus, the spread in simulated tropical Pacific SST is narrower.

Of the 10,000 trend members, we condition the top 0.5% with the lowest bias in tropical Pacific SST trends. Specifically, the bias for each trend member is measured as the root-mean-squared difference between the observed SST trends in the region where pacemaking is applied (regions bordered by dashed lines in Fig. 1). These 50 members are referred to as the PacCond ensemble. The remaining 9950 members are labeled the NonCond ensemble. While the choice of 0.5% is arbitrary, selecting the top 5% or 0.25% does not change the main results of the paper. As for the pacemaking approach, we test differences between the means of the PacCond and NonCond trend distributions using a two-tailed Student’s t-test at the 5% significance level.

### Hindcast conditioning: SMYLE hindcast simulations

We utilize the Seasonal-to-Multiyear Large Ensemble prediction system (SMYLE) hindcast simulations using CESM2^54^. The SMYLE hindcast simulations have the same horizontal resolution as the CESM2 Pacific Pacemaker simulations. The radiative forcing is largely similar to that used in CESM2 Pacemaker, but the biomass burning forcing is smoother^69^. The initial conditions are derived from the JRA55^75^ reanalysis for atmosphere. Ocean and sea ice initial conditions are obtained from the forced ocean-and-sea-ice configuration of CESM2, which uses JRA55-do^76^ as atmospheric forcing.

We use the 20-member ensemble hindcast simulations initialised on the 1st of May in each year from 1970 to 2019. We use the data from months 20 to 22 of each simulation (second DJF). The lead time needed to obtain a sufficient spread in simulated tropical Pacific SST is longer than the DePreSys3 hindcasts. This results in 20 data points each year, and we focus on the same period (DJF from 1981/92 to 2018/2019) as above. We create 10,000 bootstrapped timeseries by randomly selecting one of the 20 members each year. The rest of the procedures to create the tropical Pacific SST trend-conditioned ensembles are the same as those for the DePreSys3 hindcasts. Since the hindcast conditioning method is used in the CESM2 model, the SMYLE hindcasts effectively connect the DePreSys3 hindcast conditioning and CESM2 pacemaking.

### La Niña and El Niño years

La Niña and El Niño years for quantifying ENSO variability (Δ_*ENSO*_) are defined using ERSSTv5 DJF-mean SST anomalies, calculated with respect to the 1982–2019 climatology. The long-term trends are removed from the anomalies, and we calculate the Niño3.4 index from the detrended anomalies. La Niña and El Niño years are identified when the detrended index falls below or exceeds one standard deviation, respectively^28^. This results in 5 La Niña (1988/89, 1998/99, 1999/2000, 2007/08, 2010/11) and 6 El Niño (1982/83, 1986/87, 1991/92, 1997/1998, 2009/10, 2015/16) years. The same years are used to create La Niña and El Niño composites in PacCond and PacPace ensembles. The difference between the 5 La Niña and 6 El Niño years, i.e. La Niña minus El Niño, quantifies the impact of ENSO variability (Δ_*ENSO*_).

## Supplementary information

Supplementary Information

## Data Availability

The reanalysis data used here are available online (ERA5: https://cds.climate.copernicus.eu/cdsapp#!/dataset/reanalysis-era5-pressure-levels?tab=form, JRA3Q: https://rda.ucar.edu/datasets/ds640-1/, MERRA2: https://disc.gsfc.nasa.gov/datasets?project=MERRA-2). The GPCP data are available at https://psl.noaa.gov/data/gridded/data.gpcp.html. The CESM2-LE simulations are accessible online at https://www.cesm.ucar.edu/community-projects/lens2. The Pacific Pacemaker simulations are available at https://www.cesm.ucar.edu/working-groups/climate. The SMYLE hindcasts can be accessed at https://www.cesm.ucar.edu/working-groups/earth-system/simulations/smyle. DePreSys3 and HadGEM3-GC2 data are available upon reasonable request from the authors. The underlying code for this study is available on zenodo.org and can be accessed via this link 10.5281/zenodo.15351426.

## Code availability

The underlying code for this study is available on zenodo.org and can be accessed via this link 10.5281/zenodo.15351426.

## References

[1] Deser, C., Phillips, A. S. & Alexander, M. A. Twentieth century tropical sea surface temperature trends revisited. *Geophys. Res. Lett*. **37**, 10 (2010).

[2] Coats, S. & Karnauskas, K. Are simulated and observed twentieth century tropical pacific sea surface temperature trends significant relative to internal variability? *Geophys. Res. Lett.* **44**, 9928–9937 (2017).

[3] Wills, R. C., Dong, Y., Proistosecu, C., Armour, K. C. & Battisti, D. S. Systematic climate model biases in the large-scale patterns of recent sea-surface temperature and sea-level pressure change. *Geophys. Res. Lett.* **49**, e2022GL100011 (2022).

[4] Watanabe, M. et al. Possible shift in controls of the tropical pacific surface warming pattern. *Nature* **630**, 315–324 (2024). 38867130 10.1038/s41586-024-07452-7

[5] Seager, R. et al. Strengthening tropical pacific zonal sea surface temperature gradient consistent with rising greenhouse gases. *Nat. Clim. Change* **9**, 517–522 (2019).

[6] Lee, S. et al. On the future zonal contrasts of equatorial pacific climate: Perspectives from observations, simulations, and theories. *npj Clim. Atmos. Sci.* **5**, 82 (2022).

[7] Dong, L. & Zhou, T. The formation of the recent cooling in the eastern tropical pacific ocean and the associated climate impacts: A competition of global warming, ipo, and amo. *J. Geophys. Res. Atmos.* **119**, 11–272 (2014).

[8] Seager, R., Henderson, N. & Cane, M. Persistent discrepancies between observed and modeled trends in the tropical pacific ocean. *J. Clim.* **35**, 4571–4584 (2022).

[9] Eyring, V. et al. Overview of the coupled model intercomparison project phase 6 (cmip6) experimental design and organization. *Geosci. Model. Dev.* **9**, 1937–1958 (2016).

[10] Wang, Z., Dong, L., Song, F., Zhou, T. & Chen, X. Uncertainty in the past and future changes of tropical pacific sst zonal gradient: Internal variability versus model spread. *J. Clim.* **37**, 1465–1480 (2024).

[11] Kosaka, Y. & Xie, S.-P. Recent global-warming hiatus tied to equatorial pacific surface cooling. *Nature* **501**, 403–407 (2013). 23995690 10.1038/nature12534

[12] Andrews, T. et al. Accounting for changing temperature patterns increases historical estimates of climate sensitivity. *Geophys. Res. Lett.* **45**, 8490–8499 (2018).

[13] Dong, Y., Proistosescu, C., Armour, K. C. & Battisti, D. S. Attributing historical and future evolution of radiative feedbacks to regional warming patterns using a green’s function approach: The preeminence of the western pacific. *J. Clim.* **32**, 5471–5491 (2019).

[14] Armour, K. C. et al. Sea-surface temperature pattern effects have slowed global warming and biased warming-based constraints on climate sensitivity. *Proc. Natl Acad. Sci.* **121**, e2312093121 (2024). 38466843 10.1073/pnas.2312093121 PMC10962993

[15] Kang, J. M., Shaw, T. A., Kang, S. M., Simpson, I. R. & Yu, Y. Revisiting the reanalysis-model discrepancy in southern hemisphere winter storm track trends. *npj Clim. Atmos. Sci.* **7**, 252 (2024).

[16] Dong, B., Sutton, R. T., Shaffrey, L. & Harvey, B. Recent decadal weakening of the summer eurasian westerly jet attributable to anthropogenic aerosol emissions. *Nat. Commun.* **13**, 1148 (2022). 35241666 10.1038/s41467-022-28816-5 PMC8894405

[17] Kang, J. M., Shaw, T. A. & Sun, L. Anthropogenic aerosols have significantly weakened the regional summertime circulation in the northern hemisphere during the satellite era. *AGU Adv.* **5**, e2024AV001318 (2024). 10.1029/2024AV001318 PMC11600774 39606593

[18] Woollings, T., Drouard, M., O’Reilly, C. H., Sexton, D. M. & McSweeney, C. Trends in the atmospheric jet streams are emerging in observations and could be linked to tropical warming. *Commun. Earth Environ.* **4**, 125 (2023).

[19] Ting, M. & Yu, L. Steady response to tropical heating in wavy linear and nonlinear baroclinic models. *J. Atmos. Sci.* **55**, 3565–3582 (1998).

[20] Seager, R., Harnik, N., Kushnir, Y., Robinson, W. & Miller, J. Mechanisms of hemispherically symmetric climate variability. *J. Clim.* **16**, 2960–2978 (2003).

[21] L’Heureux, M. L. & Thompson, D. W. Observed relationships between the El Niño–Southern Oscillation and the extratropical zonal-mean circulation. *J. Clim.* **19**, 276–287 (2006).

[22] Lu, J., Chen, G. & Frierson, D. M. Response of the zonal mean atmospheric circulation to el niño versus global warming. *J. Clim.* **21**, 5835–5851 (2008).

[23] Nakamura, H., Sampe, T., Tanimoto, Y. & Shimpo, A. Observed associations among storm tracks, jet streams and midlatitude oceanic fronts. *AGU Geophys. Monogr. Ser.* **147**, 329–345 (2004).

[24] Barpanda, P. & Shaw, T. Using the moist static energy budget to understand storm-track shifts across a range of time scales. *J. Atmos. Sci.* **74**, 2427–2446 (2017).

[25] Shaw, T. A., Barpanda, P. & Donohoe, A. A moist static energy framework for zonal-mean storm-track intensity. *J. Atmos. Sci.* **75**, 1979–1994 (2018).

[26] Horel, J. D. & Wallace, J. M. Planetary-scale atmospheric phenomena associated with the Southern Oscillation. *Mon. Weather Rev.* **109**, 813–829 (1981).

[27] Trenberth, K. E. et al. Progress during toga in understanding and modeling global teleconnections associated with tropical sea surface temperatures. *J. Geophys. Res. Oceans* **103**, 14291–14324 (1998).

[28] Deser, C., Simpson, I. R., McKinnon, K. A. & Phillips, A. S. The northern hemisphere extratropical atmospheric circulation response to enso: How well do we know it and how do we evaluate models accordingly? *J. Clim.* **30**, 5059–5082 (2017).

[29] Cai, W. et al. Climate impacts of the el niño–southern oscillation on south america. *Nat. Rev. Earth Environ.* **1**, 215–231 (2020).

[30] Ropelewski, C. F. & Halpert, M. S. North American precipitation and temperature patterns associated with the El Niño/Southern Oscillation (ENSO). *Mon. Weather Rev.* **114**, 2352–2362 (1986).

[31] Trenberth, K. E., Caron, J. M., Stepaniak, D. P. & Worley, S. Evolution of el niño–southern oscillation and global atmospheric surface temperatures. *J. Geophys. Res. Atmos*. **107**, D8 AAC−5 (2002).

[32] Seager, R. et al. Mechanisms of enso-forcing of hemispherically symmetric precipitation variability. *Q. J. R. Meteorol. Soc.* **131**, 1501–1527 (2005).

[33] Ashok, K., Behera, S. K., Rao, S. A., Weng, H. & Yamagata, T. El niño modoki and its possible teleconnection. *J. Geophys. Res. Oceans* **112**, C11 (2007).

[34] Dieppois, B., Rouault, M. & New, M. The impact of enso on southern african rainfall in cmip5 ocean atmosphere coupled climate models. *Clim. Dyn.* **45**, 2425–2442 (2015).

[35] Shaw, T. A. et al. Regional climate change: consensus, discrepancies, and ways forward. *Front. Clim.* **6**, 1391634 (2024).

[36] Simpson, I. R. et al. Confronting earth system model trends with observations. *Sci. Adv.* **11**, eadt8035 (2025). 40073142 10.1126/sciadv.adt8035 PMC11900882

[37] Thomas, R., Woollings, T. & Dunstone, N. The role of internal variability in seasonal hindcast trend errors. *J. Clim.* (2025).

[38] Thorne, P. W., Lanzante, J. R., Peterson, T. C., Seidel, D. J. & Shine, K. P. Tropospheric temperature trends: history of an ongoing controversy. *Wiley Interdiscip. Rev. Clim. Change* **2**, 66–88 (2011).

[39] Mitchell, D. M., Thorne, P. W., Stott, P. A. & Gray, L. J. Revisiting the controversial issue of tropical tropospheric temperature trends. *Geophys. Res. Lett.* **40**, 2801–2806 (2013).

[40] Po-Chedley, S. et al. Natural variability contributes to model-satellite differences in tropical tropospheric warming. *Proc. Natl. Acad. Sci*.**118** https://www.pnas.org/content/118/13/e2020962118 (2021). Publisher: National Academy of Sciences. 10.1073/pnas.2020962118 PMC8020635 33753490

[41] Yang, D. et al. Role of tropical variability in driving decadal shifts in the southern hemisphere summertime eddy-driven jet. *J. Clim.* **33**, 5445–5463 (2020).

[42] Patterson, M. & O’Reilly, C. H. Climate models struggle to simulate observed north pacific jet trends, even accounting for tropical pacific sea surface temperature trends. *Geophys. Res. Lett.* **52**, e2024GL113561 (2025).

[43] Ineson, S. & Scaife, A. A. The role of the stratosphere in the european climate response to el niño. *Nat. Geosci.* **2**, 32–36 (2009).

[44] Molteni, F. & Brookshaw, A. Early- and late-winter ENSO teleconnections to the euro-atlantic region in state-of-the-art seasonal forecasting systems. *Clim. Dyn.* **61**, 2673–2692 (2023).

[45] Goyal, R., Jucker, M., Sen Gupta, A., Hendon, H. H. & England, M. H. Zonal wave 3 pattern in the southern hemisphere generated by tropical convection. *Nat. Geosci.* **14**, 732–738 (2021).

[46] Lau, N.-C. & Nath, M. J. The role of the “atmospheric bridge” in linking tropical pacific enso events to extratropical sst anomalies. *J. Clim.* **9**, 2036–2057 (1996).

[47] Qiu, W., Collins, M., Scaife, A. A. & Santoso, A. Tropical pacific trends explain the discrepancy between observed and modelled rainfall change over the americas. *npj Clim. Atmos. Sci.* **7**, 201 (2024).

[48] Mitchell, D. M., Lo, Y. T. E., Seviour, W. J. M., Haimberger, L. & Polvani, L. M. The vertical profile of recent tropical temperature trends: Persistent model biases in the context of internal variability. *Environ. Res. Lett.* **15**, 1040b4 (2020).

[49] Keil, P., Schmidt, H., Stevens, B. & Bao, J. Variations of tropical lapse rates in climate models and their implications for upper-tropospheric warming. *J. Clim.* **34**, 9747–9761 (2021).

[50] Kamae, Y. et al. Recent slowdown of tropical upper tropospheric warming associated with pacific climate variability. *Geophys. Res. Lett.* **42**, 2995–3003 (2015).

[51] Seabrook, M. et al. Opposite Impacts of Interannual and Decadal Pacific Variability in the Extratropics. *Geophys. Res. Lett.* **50**, e2022GL101226 (2023).

[52] Enfield, D. B. & Mayer, D. A. Tropical Atlantic sea surface temperature variability and its relation to El Niño-Southern Oscillation. *J. Geophys. Res. Oceans* **102**, 929–945 (1997).

[53] Xie, S.-P. et al. Indian Ocean Capacitor Effect on Indo-Western Pacific Climate during the Summer following El Niño. *J. Clim.* **22**, 730–747 (2009).

[54] Yeager, S. G. et al. The seasonal-to-multiyear large ensemble (smyle) prediction system using the community earth system model version 2. *Geosci. Model. Dev.* **15**, 6451–6493 (2022).

[55] Wallace, J. M. & Gutzler, D. S. Teleconnections in the Geopotential Height Field during the Northern Hemisphere Winter. *Mon. Weather Rev.* **109**, 784–812 (1981).

[56] Dunstone, N. et al. Skilful predictions of the winter north atlantic oscillation one year ahead. *Nat. Geosci.* **9**, 809–814 (2016).

[57] Garreaud, R. D. et al. The Central Chile Mega Drought (2010-2018): A climate dynamics perspective. *Int. J. Climatol.* **40**, 421–439 (2020).

[58] Sun, X. et al. Enhanced jet stream waviness induced by suppressed tropical pacific convection during boreal summer. *Nat. Commun.* **13**, 1288 (2022). 35277484 10.1038/s41467-022-28911-7 PMC8917179

[59] Collins, M. El niño-or la niña-like climate change? *Clim. Dyn.* **24**, 89–104 (2005).

[60] Dong, Y., Armour, K. C., Battisti, D. S. & Blanchard-Wrigglesworth, E. Two-way teleconnections between the southern ocean and the tropical pacific via a dynamic feedback. *J. Clim.* **35**, 6267–6282 (2022).

[61] Li, Z. & Ding, Q. A global poleward shift of atmospheric rivers. *Sci. Adv.* **10**, eadq0604 (2024). 39392886 10.1126/sciadv.adq0604 PMC11468922

[62] Huang, B. et al. Extended reconstructed sea surface temperature, version 5 (ersstv5): upgrades, validations, and intercomparisons. *J. Clim.* **30**, 8179–8205 (2017).

[63] Gelaro, R. et al. The modern-era retrospective analysis for research and applications, version 2 (merra-2). *J. Clim.* **30**, 5419–5454 (2017). 10.1175/JCLI-D-16-0758.1 PMC6999672 32020988

[64] Hersbach, H. et al. The era5 global reanalysis. *Q. J. R. Meteorol. Soc.* **146**, 1999–2049 (2020).

[65] Kosaka, Y. et al. The jra-3q reanalysis. *J. Meteor. Soc. Jpn.* **102**, 49–109 (2024).

[66] Adler, R. F. et al. The global precipitation climatology project (gpcp) monthly analysis (new version 2.3) and a review of 2017 global precipitation. *Atmosphere* **9**, 138 (2018). 30013797 10.3390/atmos9040138 PMC6043897

[67] Huffman, G. J. et al. The new version 3.2 global precipitation climatology project (gpcp) monthly and daily precipitation products. *J. Clim.* **36**, 7635–7655 (2023).

[68] Danabasoglu, G. et al. The community earth system model version 2 (cesm2). *J. Adv. Model. Earth Syst.* **12**, e2019MS001916 (2020).

[69] Rodgers, K. B. et al. Ubiquity of human-induced changes in climate variability. *Earth Syst. Dyn.* **12**, 1393–1411 (2021).

[70] Williams, K. et al. The met office global coupled model 2.0 (gc2) configuration. *Geosci. Model. Dev.* **88**, 1509–1524 (2015).

[71] Senior, C. A. et al. Idealized climate change simulations with a high-resolution physical model: Hadgem3-gc2. *J. Adv. Model. Earth Syst.* **8**, 813–830 (2016).

[72] Dee, D. P. et al. The era-interim reanalysis: Configuration and performance of the data assimilation system. *Q. J. R. Meteorol. Soc.* **137**, 553–597 (2011).

[73] Smith, D. M. et al. Earth’s energy imbalance since 1960 in observations and cmip5 models. *Geophys. Res. Lett.* **42**, 1205–1213 (2015). 26074649 10.1002/2014GL062669 PMC4459179

[74] Rayner, N. et al. Global analyses of sea surface temperature, sea ice, and night marine air temperature since the late nineteenth century. *J. Geophys. Res. Atmos*. **108**, D14 (2003).

[75] Kobayashi, S. et al. The jra-55 reanalysis: General specifications and basic characteristics. *J. Meteor. Soc. Jpn.* **93**, 5–48 (2015).

[76] Tsujino, H. et al. Jra-55 based surface dataset for driving ocean–sea-ice models (jra55-do). *Ocean Model.* **130**, 79–139 (2018).

