# Supplementary material for: Robust impact of tropical Pacific SST trends on global and regional circulation in boreal winter
Source: NPJ Clim Atmos Sci. 2025 Aug 27;8(1):315. doi: 10.1038/s41612-025-01192-9 (PMC12380599; doi:10.1038/s41612-025-01192-9)
Supplement: Supplementary file 1 — Supplementary Information [file 41612_2025_1192_MOESM1_ESM.pdf]

Supplementary Materials for: Robust impact of tropical Pacific SST trends on global and regional circulation in boreal winter

Joonsuk M. Kang[1†*], Rhidian Thomas[2,3†]
Nick Dunstone[4], Tiffany A. Shaw[1], Tim Woollings[2]

[1]Department of the Geophysical Sciences, The University of Chicago
[2]Atmospheric, Oceanic and Planetary Physics, University of Oxford
[3]National Centre for Atmospheric Science, University of Reading
[4]Met Office Hadley Centre

[†]These authors contributed equally to this work.
[*]Corresponding author
Email: jmkang@uchicago.edu

Contents

Figures S1 –S10.

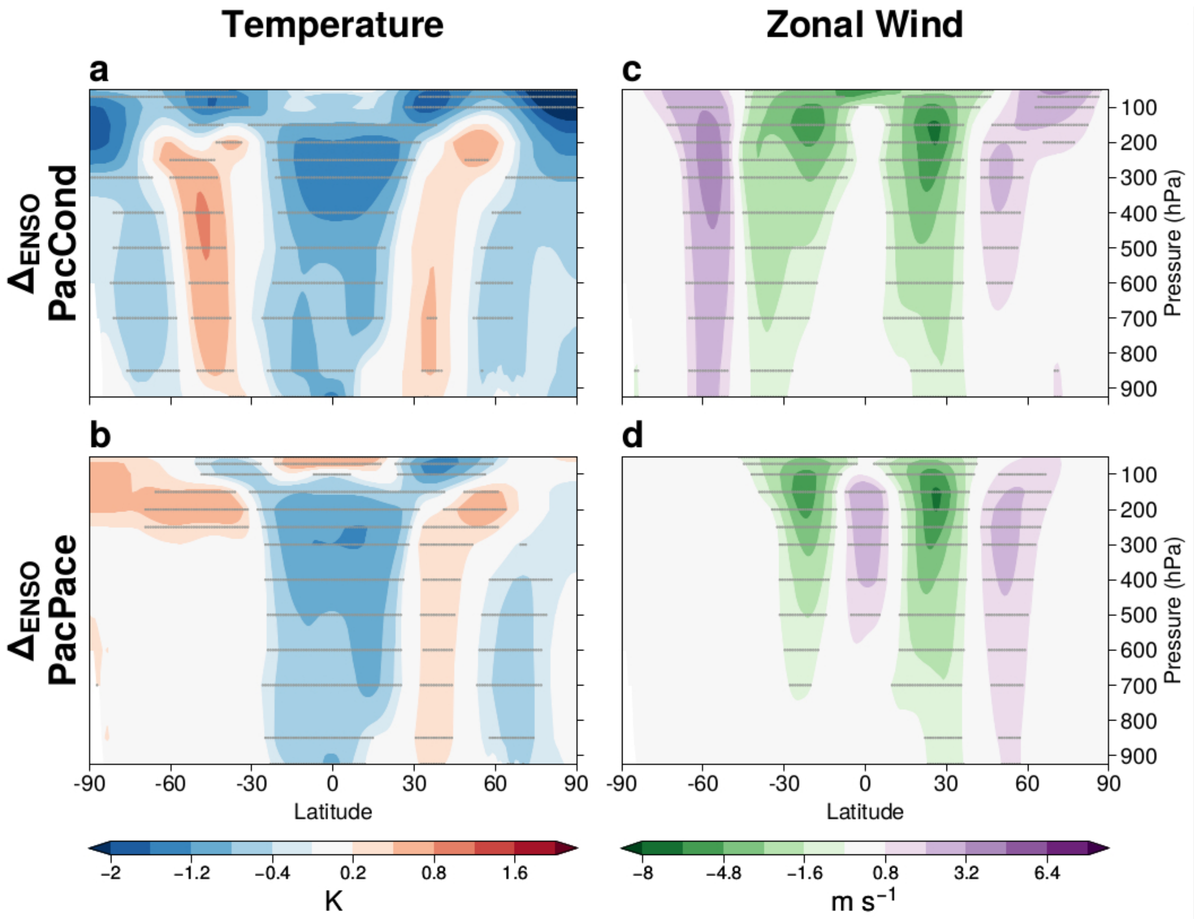

Figure S1: **Zonal-mean Δ_{ENSO} for temperature and zonal wind in the Pacific ensembles.** Similar results to Figs. 2c and f, but from the (a, c) PacCond and (b, d) PacPace ensembles.

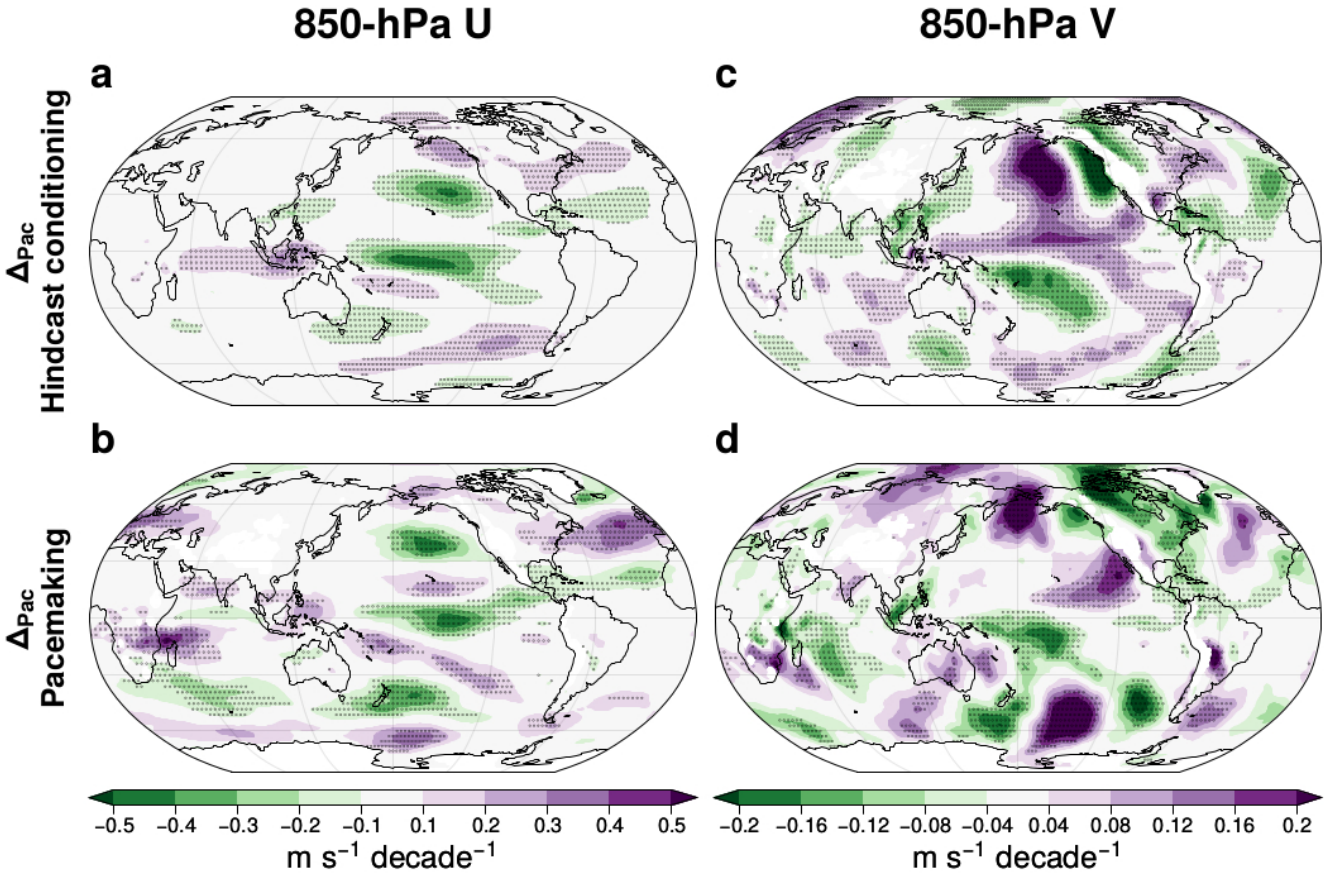

Figure S2: **Robust regional circulation impacts of constraining Pacific SST trends through hindcast conditioning and pacemaking** Similar results to Figs. 3a–d, but for 850-hPa (a, b) zonal and (c, d) meridional winds.

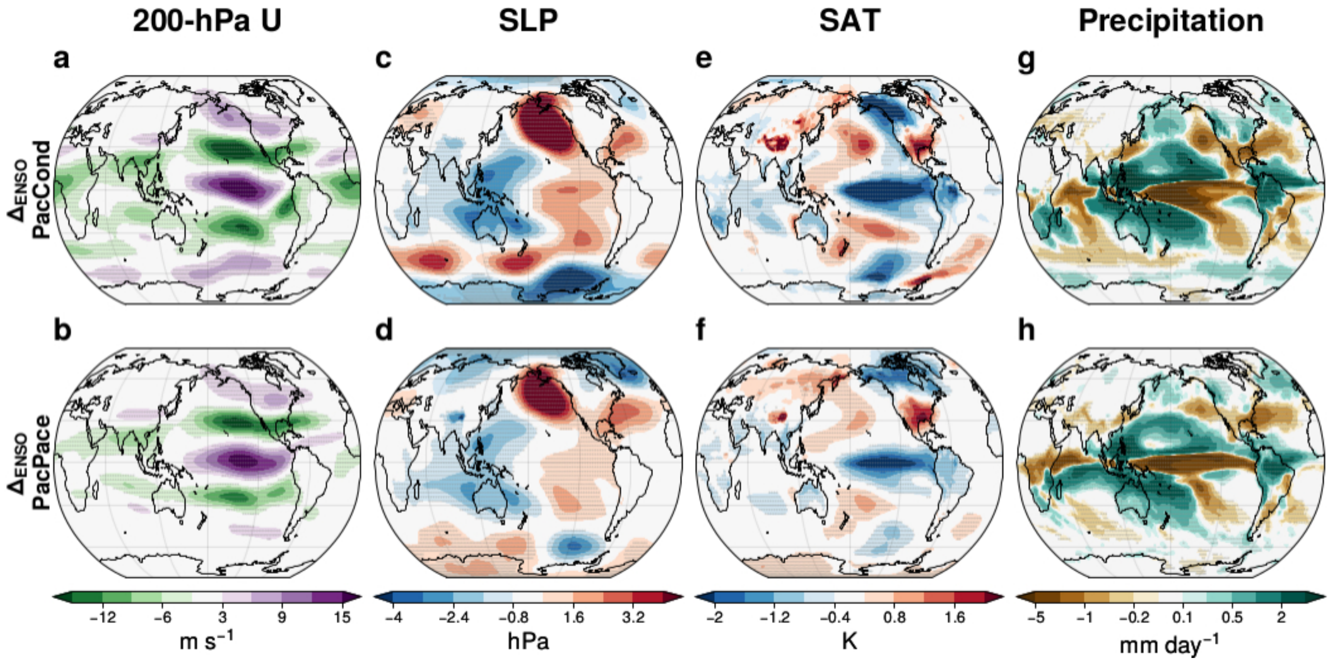

Figure S3: **Regional Δ_{ENSO} in the Pacific ensembles.** Similar results to Figs. 4c and f, but for (a, b) 200-hPa zonal wind, (c, d) SLP, (e, f) SAT, and (g, h) precipitation for (a, c, e, g) PacCond and (b, d, f, h) PacPace.

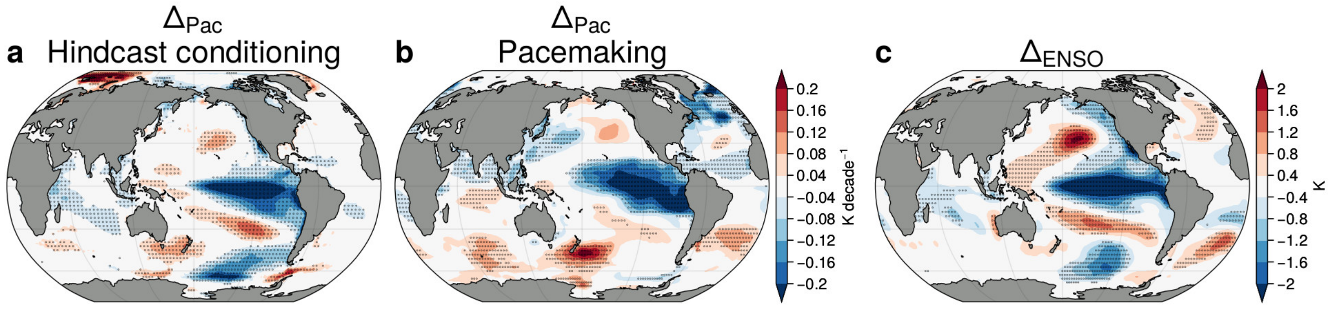

Figure S4: **Robust global SST trend impacts of constraining tropical Pacific SST trends using hindcast conditioning and pacemaking resemble ENSO variability**. Similar results to Figs. 4a-c, but for SST.

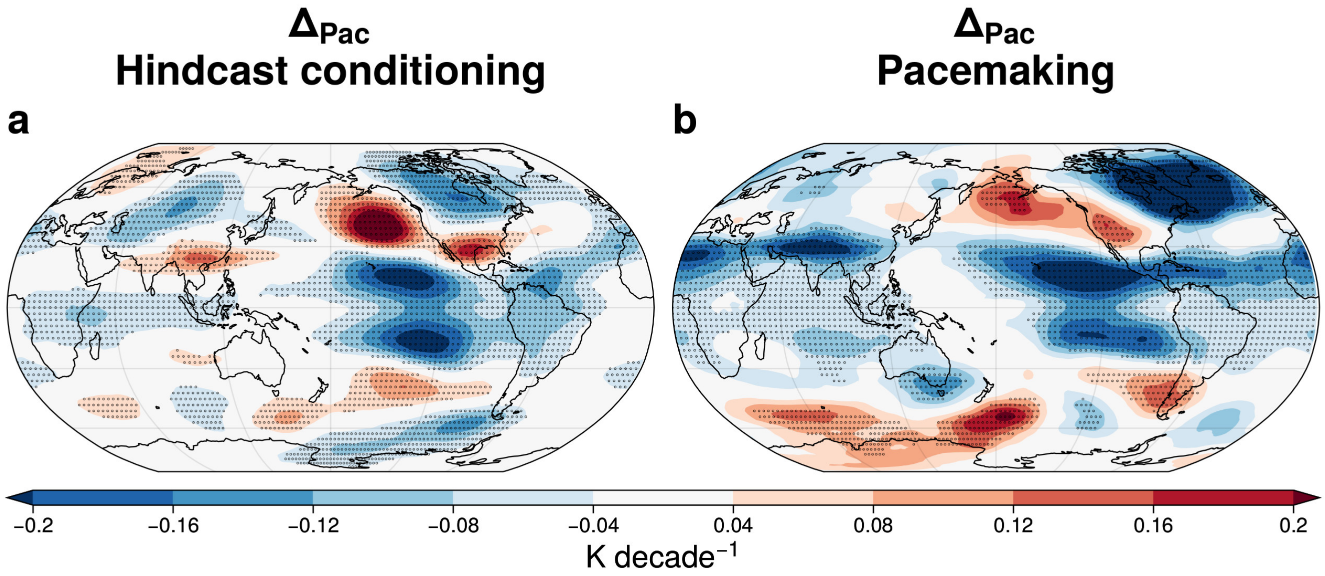

Figure S5: **Robust impacts of constraining Pacific SST trends on mid tropospheric temperature through hindcast conditioning and pacemaking.** Similar results to Figs. 3a and b, but for 500-hPa temperature.

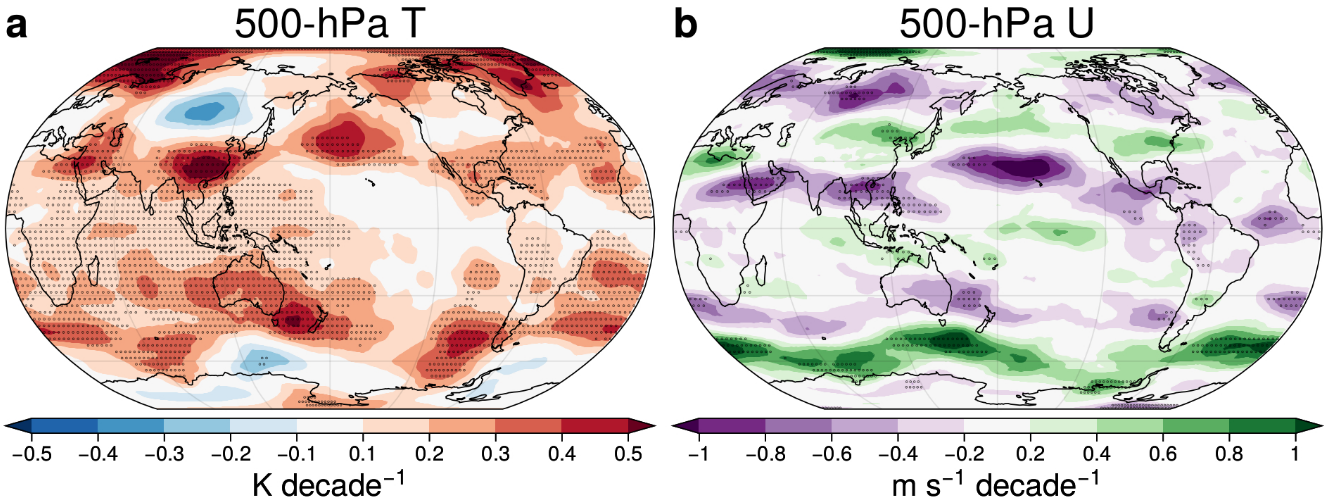

Figure S6: **Regional patterns of reanalysis circulation trends.** Spatial patterns of 500-hPa (a) temperature and (b) zonal wind trends during DJF from 1981/82 to 2018/19 in ERA5 reanalysis. Statistically significant trends at the 95% level are stippled.

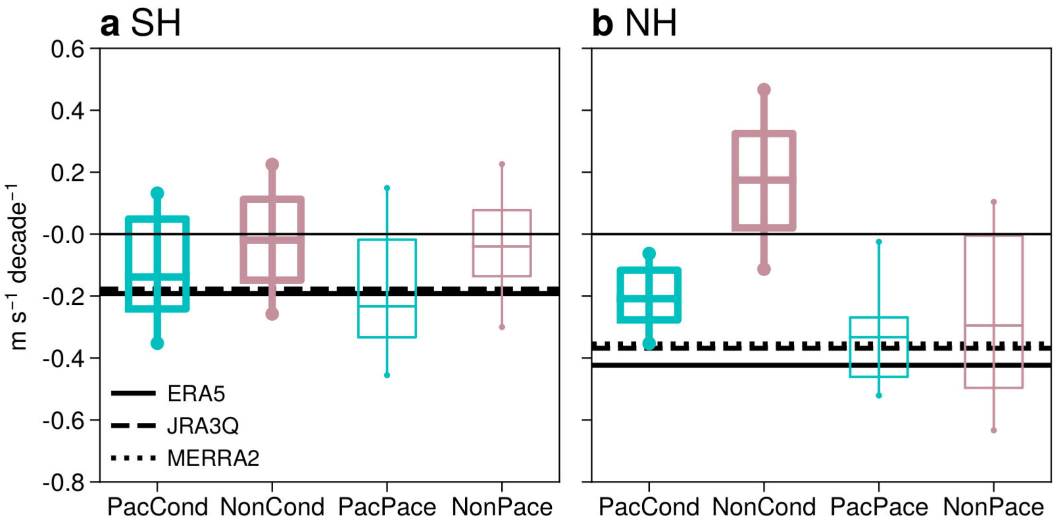

Figure S7: **Constraining Pacific SST trends improves coupled model subtropical jet trends.** Similar results to Fig. 6, but for subtropical jet trends. The subtropical jet trends are defined as the average between 400 and 200 hPa for 30–45°S in the SH and 15–30°N in the NH.

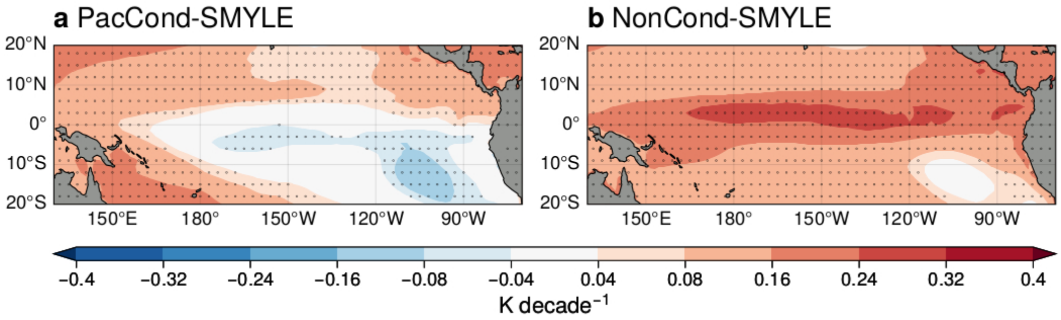

Figure S8: **Observed tropical Pacific SST trends can be captured by conditioning SMYLE hindcast simulations.** Tropical Pacific DJF SST trends (1981/82-2018/19) in SMYLE (a) PacCond and (b) NonCond ensemble mean. Stipples indicate statistically significant trends at the 5% significance level.

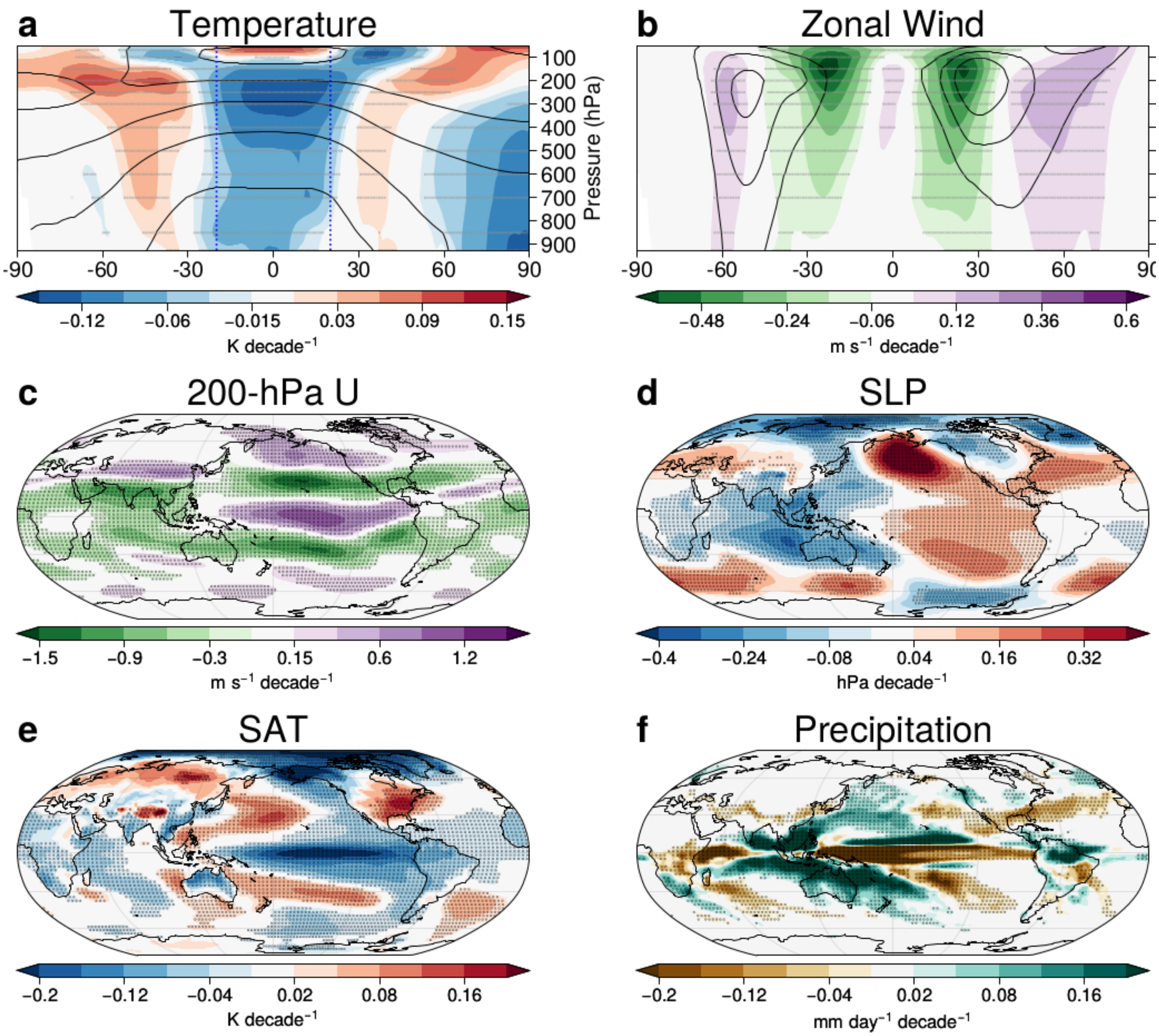

Figure S9: **Impacts of constraining Pacific SST trends through hindcast conditioning using the SMYLE simulations are consistent with other methods.** Similar results to Figs. 2–4, but hindcast conditioning is applied to SMYLE simulations.

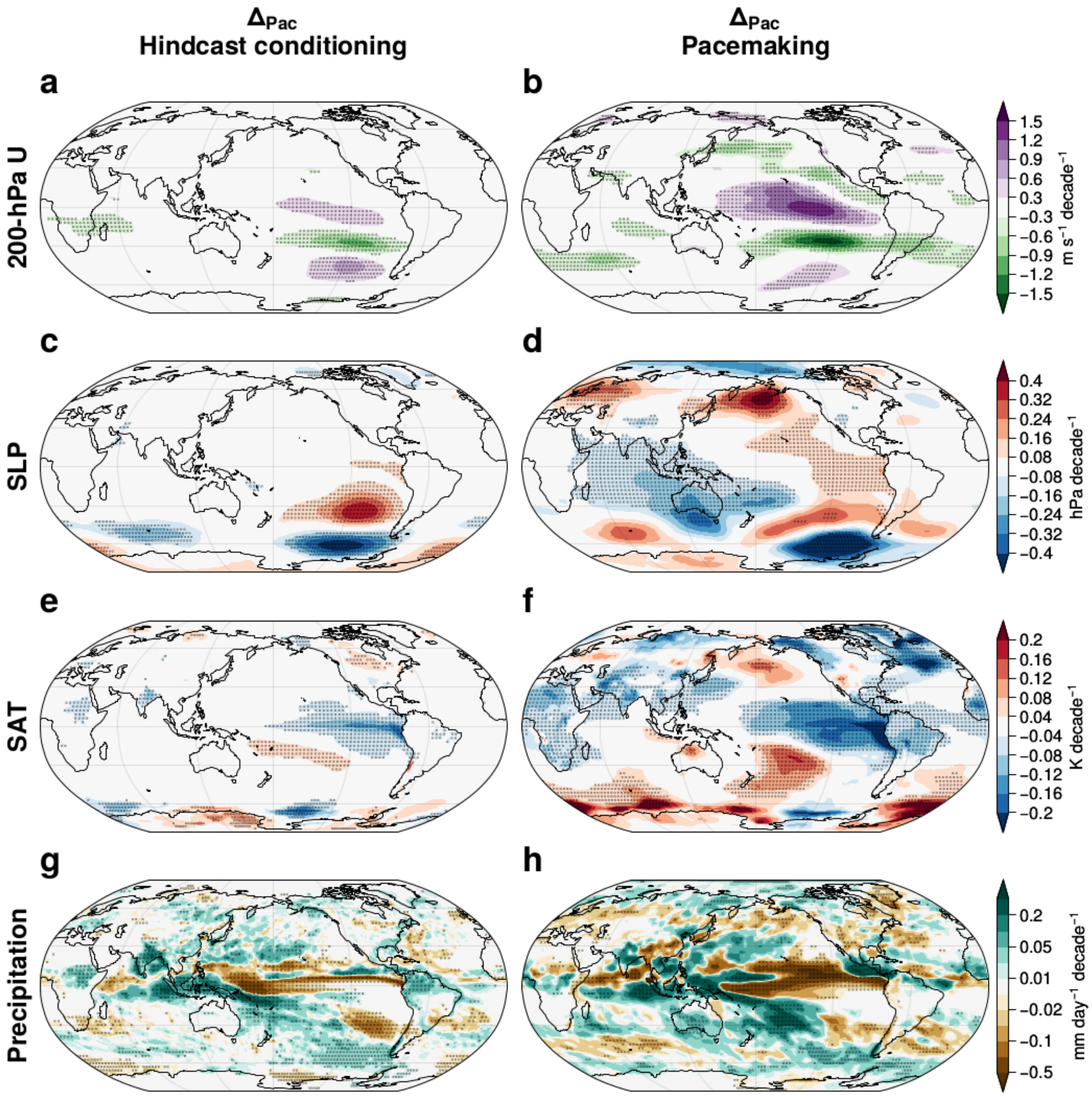

Figure S10: **Constraining tropical Pacific SST trends during JJA has robust impacts on South Pacific circulation trends.** Similar results to Figs. 3 and 4, but for JJA.
